# Sulfur-containing spiroketals from *Breynia disticha* and evaluations of their anti-inflammatory effect

**DOI:** 10.3762/bjoc.19.117

**Published:** 2023-10-19

**Authors:** Ken-ichi Nakashima, Naohito Abe, Masayoshi Oyama, Hiroko Murata, Makoto Inoue

**Affiliations:** 1 Laboratory of Medicinal Resources, School of Pharmacy, Aichi Gakuin University, 1-100 Kusumoto-cho, Chikusa-ku, Nagoya, Aichi 464-8650, Japan; 2 Laboratory of Pharmacognosy, Department of Bioactive Molecules, Gifu Pharmaceutical University, 1-25-4 Daigaku-nishi, Gifu 501-1196, Japan; 3 retired, formerly Faculty of Pharmaceutical Sciences, Setsunan University, 45-1 Nagaotoge-cho, Hirakata, Osaka, 573-0101, Japan

**Keywords:** anti-inflammatory, *Breynia disticha*, RAW 264.7 cells, sesquiterpenoids, sulfur-containing compounds

## Abstract

*Breynia* spp. are a key source of sulfur-containing spiroketal glycosides with potential anti-inflammatory activity. In this study, three new sulfur-containing spiroketals – breynin J (**1**), epibreynin J (**2**), and probreynogenin (**3**) – along with four known compounds – probreynin I (**4**), phyllaemblic acid (**5**), breynin B (**6**), and epibreynin B (**7**) – were isolated from the roots of *Breynia disticha*. The structures of compounds **1**–**7** were elucidated by extensive 1D and 2D NMR spectroscopic analyses, including 1D total correlation spectroscopy (TOCSY), HSQC, HMBC, double quantum-filtered (DQF)-COSY, heteronuclear two-bond correlation (H2BC), and HSQC-TOCSY experiments, as well as high-resolution electrospray ionization HRESIMS analysis, and quantum chemical electronic CD calculations. Furthermore, the absolute configurations of sugar residues were determined by derivatization of the hydrolysates with ʟ-cysteine methyl ester and *o*-tolyl isothiocyanate followed by HPLC analysis. The anti-inflammatory effects of the isolated compounds were evaluated based on the mRNA levels of proinflammatory cytokines in lipopolysaccharide (LPS)-stimulated RAW 264.7 murine macrophage cells. Compounds **1**, **2**, **6**, and **7** inhibited the increase in interleukin (IL)-1β and IL-6 mRNA levels stimulated by LPS. Moreover, the most potent compound **7** was found to significantly inhibit the production of IL-1β and IL-6 proteins, as revealed by the analysis of culture supernatants.

## Introduction

*Breynia disticha* J.R.Forst. & G.Forst. (Phyllanthaceae), commonly known as snow bush, is a tropical shrub native to New Caledonia and Vanuatu in the Western Pacific but has been naturalized in many regions globally. Investigations of the phytochemical compositions of several *Breynia* spp. have revealed sulfur-containing spiroketal glycosides [1–3], flavan-3-ol glycosides [4], alkaloids [5], and phenolic glycosides [5–6]. Notably, sulfur-containing spiroketal glycosides, represented by breynins and epibreynins, have characteristic sesquiterpenoid-derived structures. The sesquiterpenoid phyllaemblic acid and its glycosides, which are predicted to be biosynthetic precursors of breynins and epibreynins, have been isolated from several species of the genera *Phyllanthus* and *Glochidion* (both Phyllanthaceae) [7–10]. Nonetheless, to date, sulfur-containing derivatives have only been identified in the genus *Breynia*.

*Breynia* spp. have traditionally been used as anti-inflammatory medicines for intestinal and stomach inflammation, asthma, eczema, meningitis, and sore throat [11]. Furthermore, epibreynin D, a major sulfur-containing spiroketal in *Breynia fruticosa*, has been reported to have anti-arthritis activity against complete Freund's adjuvant-induced arthritis in rats [12]. However, the molecular mechanisms of these anti-inflammatory effects have not been investigated.

**Figure 1:**
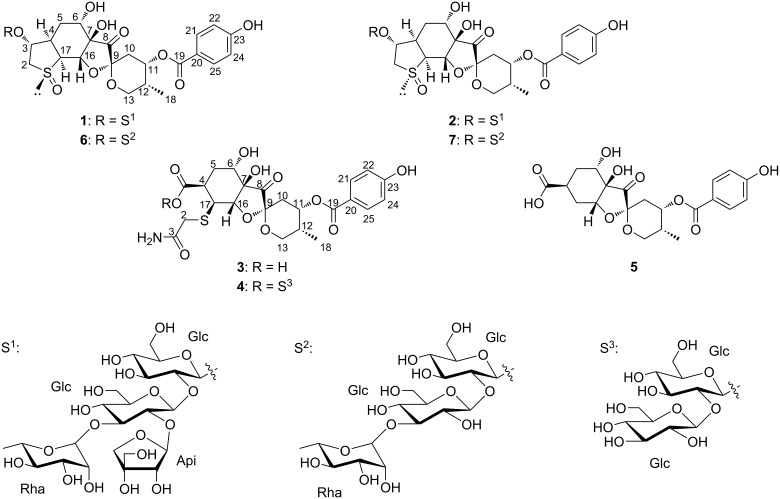
Structures of compounds **1**–**7**.

In our ongoing search for bioactive natural products, we have isolated three new spiroketals – breynin J (**1**), epibreynin J (**2**), and probreynogenin (**3**) – and four known spiroketals – probreynin I (**4**) [13], phyllaemblic acid (**5**) [10], breynin B (**6**) [2], and epibreynin B (**7**) [2] – from the roots of *B. disticha*, a species that has not previously been chemically investigated (Figure 1). Herein, we describe the isolation and structural elucidation of new compounds **1**–**3** and known spiroketal **4**, which has not been fully characterized in previous reports [13]. Furthermore, the anti-inflammatory effects of compounds **1**–**3**, **6**, and **7** are evaluated using RAW 264.7 murine macrophage cells.

## Results and Discussion

*B. disticha* roots were extracted with methanol (MeOH), and the extract suspended in water was sequentially partitioned with ethyl acetate and *n*-butanol. Breynin J (**1**), epibreynin J (**2**), and probreynin I (**4**) together with known compounds **6** and **7** were isolated from the *n*-butanol fraction (Figure 1). Probreynogenin (**3**) and known compound **5** were obtained from the ethyl acetate fraction (Figure 1). The structures of known compounds **5**–**7** were identified based on ^1^H and ^13^C NMR data [2–3,10].

**Figure 2:**
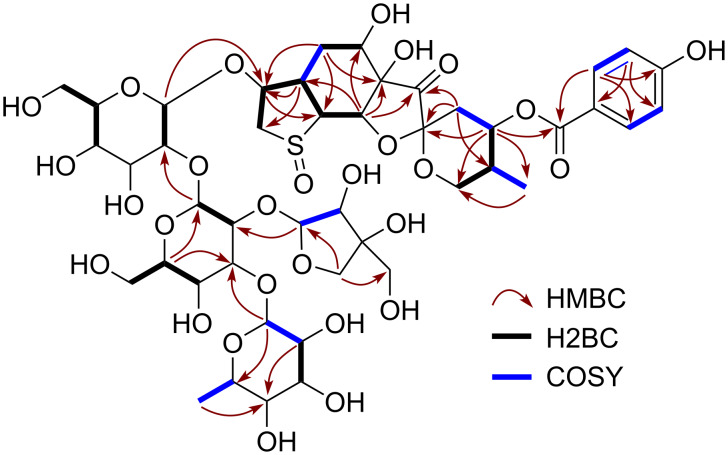
Key HMBC (red arrows), H2BC (black bold lines), and COSY (blue bold lines) correlations in **1** and **2**.

Breynin J (**1**) was isolated as an amorphous, colorless powder. The HRESIMS spectrum exhibited a sodium adduct ion peak at *m*/*z* 1107.3177, consistent with a molecular formula of C_45_H_64_O_28_SNa (calcd 1107.3197). The IR spectrum showed absorption peaks corresponding to hydroxy groups (ν_max_ = 3414 cm^−1^) and carbonyl groups (ν_max_ = 1782 and 1695 cm^−1^). The ^1^H NMR signals (Table 1) were characteristic of a breynogenin moiety, namely, a methyl group [δ_H_ 0.92 (d, *J* = 6.9 Hz, 3H, H_3_-18)], four methylene groups [δ_H_ 3.42 (br d, *J* = 15.6 Hz, 1H, H-2a), 3.70 (dd, *J* = 4.5, 15.6 Hz, 1H, H-2b), 1.70 (br d, *J* = 13.7 Hz, 1H, H-5α), 1.25 (t, *J* = 13.7 Hz, 1H, H-5β); 2.12 (d, *J* = 3.7 Hz, 2H, H_2_-10); and 3.97 (t, *J* = 10.5 Hz, 1H, H-13α), 3.65 (1H, m, H-13β)], and seven sp^3^ methines. In addition, signals corresponding to a *p*-hydroxybenzoate group [δ_H_ 8.02 (d, *J* = 8.9 Hz, 2H, H-21, 25), 6.94 (d, *J* = 8.9 Hz, 2H, H-22, 24)] were observed. The ^13^C NMR spectrum (Table 1) suggested the presence of a breynogenin-α-*S*-oxide moiety, similar to that in breynins B, D, G, and I [2], with the observed resonances including a carbonyl group [δ_C_ 212.3 (C-8)], a spiroketal center [δ_C_ 100.6 (C-9)], and a *p*-hydroxybenzoate [δ_C_ 167.6 (C-19), 122.7 (C-20), 133.2 (C-21, 25), 116.7 (C-22, 24), 163.4 (C-23)]. The presence of a breynogenin-α-*S*-oxide moiety was further evidenced by HSQC, HMBC, and double quantum filtered (DQF)-COSY correlations (Figure 2). The spin–spin coupling constants (SSCCs) in the ^1^H NMR spectrum and the rotating-frame Overhauser effects (ROEs) between H-2β/H-3, H-2β/H-5β, H-3/H-4, H-3/5β, H-4/H-5α, H-4/H-17, H-5α/H-6, H-5β/H-6, H-11/H-12, H-12/H-13β, H-16/H-22(24), and H-17/H-21(25) in 1D and 2D ROE spectroscopy (ROESY) suggested that the relative configuration of the aglycone in **1** is identical to that in breynin B (**6**).

**Table 1:** ^1^H and ^13^C NMR data for the aglycone moieties of **1**–**4**.^a^

no.	**1**			**2**			**3**			**4**	
						
	δ_C_	δ_H_ (mult, *J*)		δ_C_	δ_H_ (mult, *J*)		δ_C_	δ_H_ (mult, *J*)		δ_C_	δ_H_ (mult, *J*)

2	56.2	3.42 (br d, 15.6)		61.7	4.46 (d, 14.6)		37.3	3.32 (d, 15.6)		36.96	3.24 (d, 16.0)
		3.70 (dd, 4.5, 15.6)			3.10 (dd, 4.1, 15.1)			3.44 (d, 15.6)			3.64 (d, 16.0)
3	87.7	4.53 (br d, 4.5)		87.4	4.73 (d, 4.1)		175.0			174.7	
4	38.7	3.10 (br d, 13.7)		39.9	3.15 (m)		36.6	3.28 (ddd, 3.1, 5.5, 14.9)		37.03	3.37 (br d, 12.4)
5	28.9	1.70 (br d, 13.7)		28.4	1.87 (m)		28.3	1.88 (dt, 2.3, 13.7)		27.4	1.85 (br d, 14.6)
		1.25 (t, 13.7)			1.80 (br t, 13.0)			1.98 (m)			2.07 (br t, 13.0)
6	70.3	3.94 (br s)		71.5	3.94 (m)		71.7	3.90 (br s)		71.3	3.92 (br s)
7	75.2			73.3			76.3			76.0	
8	212.3			209.9			213.3			212.9	
9	100.6			101.1			100.6			100.7	
10	32.5	2.12 (d, 3.7)		32.6	2.13 (d, 3.2)		32.7	1.98 (m)		33.0	2.00 (dd, 2.7, 14.6)
								2.23 (dd, 3.2, 15.4)			2.18 (dd, 3.2, 15.1)
11	69.8	5.44 (m)		69.8	5.42 (q, 3.2)		70.3	5.28 (q, 3.2)		70.0^b^	5.31 (br s)
12	34.3	2.19 (m)		34.2	2.20 (m)		34.3	2.15 (m)		34.5	2.13 (m)
13	64.1	3.97 (t, 10.5)		64.0	4.06 (t, 11.0)		63.5	4.04 (t, 11.0)		63.5	4.08 (t, 11.4)
		3.65 (m)			3.69 (m)			3.57 (dd, 4.6, 11.4)			3.58 (d, 5.5, 12.4)
16	74.9	4.89 (m)		74.4	4.90 (m)		79.9	4.52 (t, 1.8)		80.2	4.60 (t, 1.4)
17	71.3	3.99 (d, 5.5)		62.3	3.95 (m)		46.1	3.73 (t, 2.3)		45.3	3.74 (br s)
18	12.9	0.92 (d, 6.9)		12.9	0.91 (d, 6.9)		13.1	0.88 (d, 6.9)		13.2	0.85 (d, 6.9)
19	167.6			167.5			168.1			167.6	
20	122.7			122.8			122.8			123.0	
21, 25	133.2	8.02 (d, 8.9)		133.3	8.01 (d, 8.7)		133.0	7.99 (d, 8.7)		133.4	8.07 (d, 8.9)
22, 24	116.7	6.94 (d, 8.9)		116.5	6.88 (d, 8.7)		116.4	6.90 (d, 8.7)		116.9	6.98 (d, 8.9)
23	163.4			163.4			163.4			163.2	
COOH							177.1			172.6	

^a1^H and ^13^C NMR data were obtained at 400 and 100 MHz, respectively. All spectra were measured in CD_3_OD (δ in ppm, *J* in Hz). Overlapped signals were assigned based on the DQF-COSY, HSQC, HMBC, 1D-TOCSY, H2BC, and HSQC-TOCSY. ^b^Overlapping signals with C-4′′ of the sugar moiety.

The remaining NMR resonances (Table 2) were attributed to a tetrasaccharide based on the observation of four anomeric methine signals at δ_C_ 103.2 (C-1′), 104.5 (C-1′′), 103.3 (C-1′′′), and 111.9 (C-1′′′′). Because the complex overlap of the glycosyl group signals complicated the HSQC, HMBC, and DQF-COSY analyses, 1D total correlation spectroscopy (TOCSY), HSQC-TOCSY, and heteronuclear two-bond correlation (H2BC) experiments were also performed (Figure 2) [14–15]. The HSQC-TOCSY spectrum showed two correlation series corresponding to two ᴅ-glucopyranoses (δ_C_ 62.5, 70.0, 77.5, 77.9, 85.0, 103.2 and δ_C_ 61.5, 69.3, 76.7, 82.9, 85.5, 104.5). The signal at δ_C_ 85.0 was assigned to C-2′ based on the H2BC correlation with the anomeric proton at δ_H_ 4.57 (d, *J* = 7.8 Hz, 1H, H-1′), which corresponded to the carbon at δ_C_ 103.2 (C-1′). Furthermore, the 1D-TOCSY experiment involving irradiation at δ_H_ 2.52 (br d, *J* = 9.2 Hz, 1H, H-5′′) resulted in the excitement of resonances at δ_H_ 3.32 (m, 3H, H-2′′, 3′′, 4′′, overlapping) and 3.50 (br s, 2H, H_2_-6′′) as well as an anomeric proton resonance at δ_H_ 4.30 (d, *J* = 7.3 Hz, 1H, H-1′′), which corresponded to the carbon at δ_C_ 104.5 (C-1′′). Based on the H2BC correlations of H-1′′/C-2′′ and H-5′′/C-4′′, the signals at δ_C_ 82.9 and 69.3 were assigned to C-2′′ and C-4′′, respectively, and that at δ_C_ 85.5 was assigned to C-3′′. The HSQC, 1D-TOCSY, and HSQC-TOCSY spectra also revealed a series of carbon signals corresponding to ʟ-rhamnose at δ_C_ 17.9, 70.5, 72.0, 72.1, 73.7, and 103.3. The remaining five carbon signals [an anomeric methine at δ_C_ 111.9 (C-1′′′′), a methine at δ_C_ 77.5 (C-2′′′′), two methylenes at δ_C_ 64.8 (C-5′′′′) and 74.5 (C-4′′′′), and a nonprotonated carbon at δ_C_ 79.3 (C-3′′′′)] were characteristic of apiofuranose. The presence of apiofuranose was also supported by the HMBC correlations of H-1′′′′/C-3′′′′, C-4′′′′ and H_2_-4′′′′/C-5′′′′, C-2′′′′ as well as the H2BC correlation of H-1′′′′/C-2′′′′. The linkages in the sugar moiety were determined based on the HMBC correlations of H-1′/C-3, H-1′′/C-2′, H-1′′′/C-3′′′, and H-1′′′′/C-2′′ and the SSCCs of the anomeric protons.

**Table 2:** ^1^H and ^13^C NMR data for the sugar moieties of **1**, **2**, and **4**.^a^

no.	**1**			**2**			**4**	
				
	δ_C_	δ_H_ (mult, *J*)		δ_C_	δ_H_ (mult, *J*)		δ_C_	δ_H_ (mult, *J*)

Glc								

1′	103.2	4.57 (d, 7.8)		102.8	4.47 (d, 7.8)		94.0	5.78 (d, 7.8)
2′	85.0	3.38 (t, 6.0)		85.2	3.27 (t, 8.7)		84.8	3.31 (t, 8.7)
3′	77.5^b^	3.57 (t, 8.7)		77.6^c^	3.53 (t, 9.2)		77.6	3.58 (t, 9.2)
4′	70.0	3.54 (t, 9.2)		70.1	3.44 (t, 9.6)		70.4	3.66 (t, 9.6)
5′	77.9^b^	3.31 (m)		78.0	3.33 (m)		79.3	3.43 (m)
6′	62.5	3.73 (dd, 5.0, 11.4)		62.5	3.71 (dd, 5.6, 11.9)		62.2	3.83 (m)
		3.85 (dd, 2.3, 11.9)			3.89 (br d, 11.9)			3.93 (br d, 11.2)

Glc								

1′′	104.5	4.30 (d, 7.3)		104.7	4.26 (d, 6.9)		107.0	3.79 (d, 7.8)
2′′	82.9	3.32 (m)		83.0	3.35 (m)		75.9	3.07 (t, 9.2)
3′′	85.5	3.32 (m)		85.5	3.35 (m)		77.8	3.25 (t, 9.6)
4′′	69.3	3.32 (m)		69.2	3.32 (m)		70.0^d^	3.18 (t, 9.2)
5′′	76.7	2.52 (br d, 9.2)		76.6	2.44 (br d, 8.7)		77.1	2.41 (br d, 9.2)
6′′	61.5	3.50 (br s)		61.4	3.47 (br t, 2.7)		61.4	3.42 (br d, 12.3)
								3.49 (dd, 2.7, 12.3)

Rha								

1′′′	103.3	4.99 (d, 0.9)		103.3	4.98 (d, 1.4)			
2′′′	72.0^e^	4.12 (dd, 1.8, 3.2)		72.0^f^	4.11 (dd, 1.4, 3.2)			
3′′′	72.1^e^	3.67 (dd, 3.2, 9.2)		72.1^f^	3.67 (dd, 3.2, 9.2)			
4′′′	73.7	3.41 (d, 9.6)		73.7	3.41 (t, 9.6)			
5′′′	70.5	3.91 (m)		70.5	3.94 (m)			
6′′′	17.9	1.26 (d, 6.4)		17.9	1.26 (d, 6.4)			

Api								

1′′′′	111.9	5.20 (d, 4.1)		112.0	5.20 (d, 4.1)			
2′′′′	77.5^b^	3.90 (d, 4.1)		77.5^c^	3.89 (d, 4.6)			
3′′′′	79.3			79.3				
4′′′′	74.5	3.75 (d, 9.6)		74.5	3.75 (d, 9.6)			
		4.15 (d, 9.6)			4.15 (d, 9.6)			
5′′′′	64.8	3.55 (d, 11.9)		64.8	3.55 (d, 11.7)			
		3.60 (d, 11.9)			4.59 (d, 11.7)			

^a1^H and ^13^C NMR data were obtained at 400 and 100 MHz, respectively. All spectra were measured in CD_3_OD. Overlapped signals were assigned based on the DQF-COSY, HSQC, HMBC, 1D-TOCSY, H2BC, and HSQC-TOCSY. ^b,c,e,f^These assignments are interchangeable. ^d^Overlapping signals with C-11 of the aglycone moiety.

The sugars contained in **1** and their absolute configurations were confirmed by analyzing the monosaccharide mixture obtained by acid hydrolysis of **1** using the method developed by Tanaka et al. [16], in which the hydrolysates are derivatized with ʟ-cysteine methyl ester and *o*-tolyl isothiocyanate followed by HPLC analysis. For compound **1**, peaks were observed at retention times of 13.96, 20.83, and 22.16 min. The peaks at 13.96 and 22.16 min were coincident with those of the ᴅ-glucose and ʟ-rhamnose derivatives, respectively. Although the peak at 20.83 min was assumed to be attributable to apiofuranose, we were unable to obtain any standard for apiofuranose. In the reported monosaccharide identification method, the retention times of the ᴅ-glucose, ʟ-rhamnose, ᴅ-apiose, and ʟ-apiose derivatives were 17.48, 29.47, 28.57, and 15.69 min, respectively, with the ᴅ-apiose derivative having a longer retention time than the ʟ-apiose derivative [16]. Therefore, considering the retention times of the ᴅ-glucose (13.96 min) and ʟ-rhamnose (22.16 min) derivatives in this study, it is reasonable to assign the peak at 20.83 min to the ᴅ-apiose derivative. Therefore, **1** was identified as breynogenin-α-*S*-oxide 3-*O*-β-ᴅ-apiofuranosyl-(1→2)-[α-ʟ-rhamnopyranosyl-(1→3)]-β-ᴅ-glucopyranosyl-(1→2)-β-ᴅ-glucopyranoside.

Epibreynin J (**2**) was also isolated as an amorphous, colorless powder. Because the sodium adduct ion peak at *m*/*z* 1107.3178 in the HRESIMS spectrum gave the same molecular formula as that of **1** (C_45_H_64_O_28_SNa, calcd 1107.3197), compounds **1** and **2** were determined to be isomers. The 1D and 2D NMR spectroscopic data indicated that the aglycone of **2** also consisted of a breynogenin-*S*-oxide moiety. However, a comparison of the ^1^H and ^13^C NMR data for the aglycones of **1** and **2** revealed markedly different tetrahydrothiophene signals [for **2**, δ_C_ 61.7 (C-2), 87.4 (C-3), 39.9 (C-4), 62.3 (C-17)] (Table 1). According to previous literature [2], these differences indicate that the sulfoxide moieties in the aglycone of **1** and **2** are isomers. The remaining NMR resonances (Table 2) and 2D NMR spectroscopic data (Figure 2) showed that the glycosyl group of **2** was the same as that of **1**. This assignment was further supported by HPLC-based monosaccharide identification, which revealed peaks at retention times of 13.97 (ᴅ-glucose), 20.84 (ᴅ-apiose), and 22.17 (ʟ-rhamnose) min. Hence, **2** was identified as breynogenin-β-*S*-oxide 3-O-β-ᴅ-apiofuranosyl-(1→2)-[α-ʟ-rhamnopyranosyl-(1→3)]-β-ᴅ-glucoptranosyl-(1→2)-β-ᴅ-glucopyranoside.

**Figure 3:**
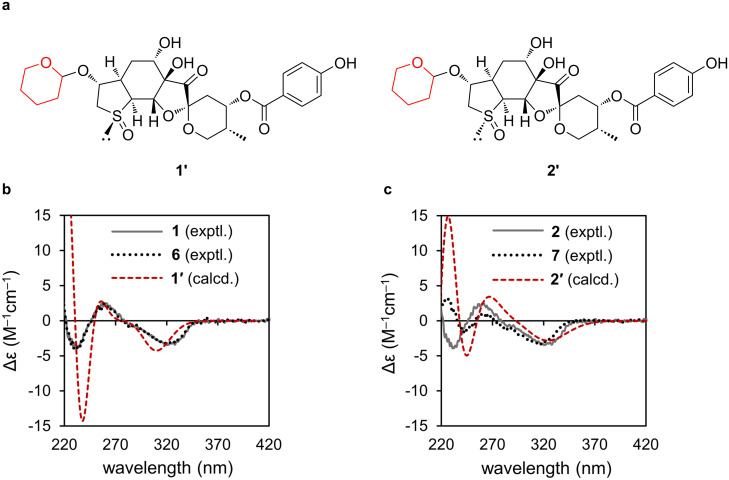
a) Simplified model structures **1′** and **2′** for GIAO and TD-DFT calculations. b) Comparison of experimental ECD spectra of **1** (gray solid line) and **6** (black dotted line) with the Boltzmann-weighted spectra computed for **1′** (red dashed line). c) Comparison of the experimental ECD spectra of **2** (gray solid line) and **7** (black dotted line) with the Boltzmann-weighted spectra computed for **2′** (red dashed line).

Furthermore, we investigated the absolute configurations of the aglycones of **1** and **2** as well as those of breynin B (**6**) and epibreynin B (**7**) by comparing their experimental electronic CD (ECD) curves with the calculated ECD curves of molecular models **1′** and **2′**, in which the glucosyl units were replaced with 2-hydroxytetrahydropyran to reduce computational costs (Figure 3a). The calculated ECD curve for **1′** agreed well with the experimental ECD spectra of **1** and **6**, indicating that the aglycones of these compounds had an absolute configuration of 1*R*,3*R*,4*R*,6*S*,7*R*,9*S*,11*S*,12*R*,16*S*,17*S* (Figure 3b). Similarly, the absolute configurations of compounds **2** and **7** were determined to be 1*S*,3*R*,4*R*,6*S*,7*R*,9*S*,11*S*,12*R*,16*S*,17*S* (Figure 3c).

**Figure 4:**
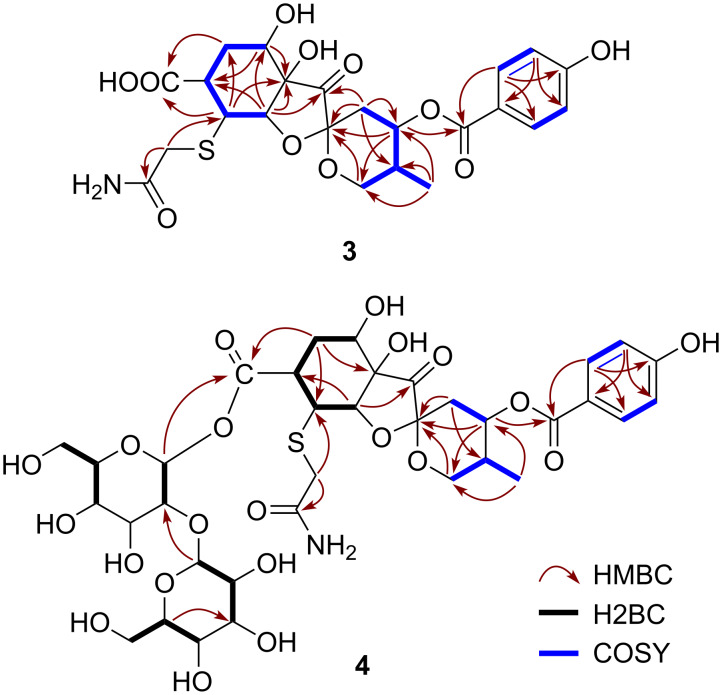
Key HMBC (red arrows), H2BC (black bold lines), and COSY (blue bold lines) correlations in compounds **3** and **4**.

**Figure 5:**
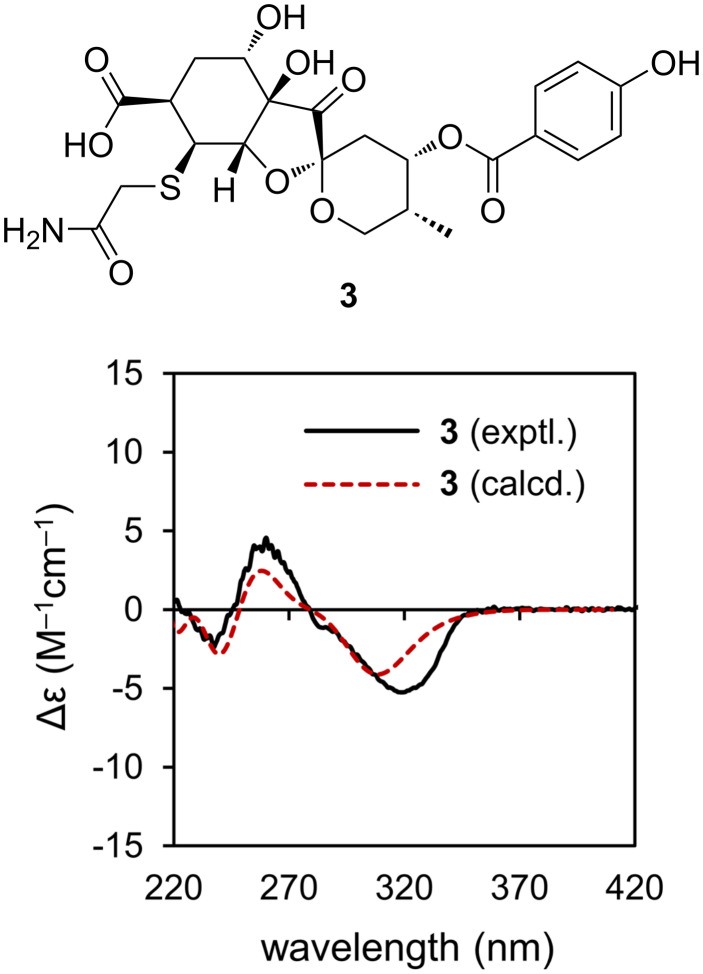
Comparison of experimental (black solid line) and calculated (red dashed line) ECD spectra of **3**.

Probreynogenin (**3**) was isolated as an amorphous, colorless solid. The molecular formula of **3** was determined to be C_23_H_27_NO_11_S based on the sodium adduct ion peak at *m*/*z* 548.1197 [M + Na]^+^ (calcd 548.1197) observed by HRESIMS. The ^1^H NMR spectrum (Table 1) showed no sugar moiety signals, but the characteristic signals of a methyl group [δ_H_ 0.88 (d, *J* = 6.9 Hz, 3H, H_3_-18)] and a *p*-hydroxybenzoate moiety [δ_H_ 7.99 (d, *J* = 8.7 Hz, 2H, H-21, 25), 6.90 (d, *J* = 8.7 Hz, 2H, H-22, 24)] were detected. The ^13^C NMR spectrum (Table 1) exhibited signals corresponding to a spiro-carbon at δ_C_ 100.6 (C-9) and a ketone carbon at δ_C_ 213.3 (C-8). The HMBC correlations of H-10β/C-8, C-9, C-11, C-12; H-11/C-9, C-13; H_2_-13/C-9; H-16/C-7, C-8; and H_3_-18/C-11, C-12, C-13 and the connectivity confirmed by DQF-COSY indicated that **3** also contained a 1,6-dioxaspiro[4,5]decane moiety with a methyl group, as in compounds **1** and **2** (Figure 4). The *p*-hydroxybenzoylcarbonyl group was connected to C-11, as evidenced by the HMBC correlation of H-11/C-19. The DQF-COSY spectrum revealed a C-16/C-17/C-3/C-4/C-5 linkage, which was confirmed by the HMBC correlations of H-5/C-3, H-16/C-3, and H-17/C-4. The linkage between C-6 and C-7 was supported by the HMBC correlations of H-5/C-6 and H-16/C-5. Furthermore, the presence of a carbonyl group was indicated by the HMBC correlations of both H-5 and H-17 with a carbonyl carbon (δ_C_ 177.1). Thus, the structure of compound **3** was highly similar to that of phyllaemblic acid (**5**), which was previously isolated from *Phyllanthus emblica* and *Glochidion coccineum* [9–10] as well as from *B. disticha* in this study. The major structural difference between these compounds was the substituent at the C-17 position. The molecular weight of **3** was 89 mass units larger than that of **5**, indicating that the unsubstituted C-17 in compound **5** was replaced with C_2_H_4_NOS. Based on the presences of the two carbons as a methylene carbon [δ_H_ 3.32 and 3.44 (d, *J* = 15.6 Hz, each 1H, H_2_-2); δ_C_ 37.3 (C-2)] and a carbonyl carbon [δ_C_ 175.0 (C-3)] as well as the HMBC correlations of H_2_-2 with C-3 and C-17, this substituent was identified as a (2-amino-2-oxoethyl)thio group. This substituent has been reported in several alkaloids isolated from plants in the Aristolochiaceae family [17–18]. Compound **3** had a β orientation for the sulfur-containing substituent at C-17 and the same relative configuration as **5** otherwise, as supported by the SSCC and NOE observed in the ^1^H NMR, NOESY, and *J*-resolved spectra. As the computed and experimental ECD curves of **3** were in good agreement, the absolute configuration of compound **3** was established as 4*R*,6*S*,7*R*,9*S*,11*S*,12*R*,16*S*,17*S* (Figure 5).

Probreynin I (**4**) was isolated as an amorphous, colorless solid. The sodium adduct ion peak at *m*/*z* 872.2241 in the HRESIMS spectrum indicated a molecular formula of C_35_H_47_NO_21_SNa (calcd 872.2253). A comparison with the molecular formula and ^1^H and ^13^C NMR spectroscopic data of **3** suggested that compound **4** contained two additional hexopyranose units (Table 1 and Table 2). The 1D-TOCSY spectrum showed two proton sequences corresponding to these hexopyranose units and the SSCCs indicated that both were glucopyranose. The characteristic ^1^H and ^13^C NMR signals of one anomeric proton [δ_H_ 5.78 (d, *J* = 7.8 Hz, 1H, H-1′); δ_C_ 94.0 (C-1′)] implied that compound **4** was a β-glycosyl ester. The HMBC correlation of H-1′ with a carbonyl carbon [δ_C_ 174.7 (C-3)] revealed that esterification occurred at C-3 with a glucopyranose moiety at the anomeric position (Figure 4). A β-1,3-glucosyl linkage between the two glucopyranose moieties was confirmed by the HMBC correlation of H-1′′/C-2′ and the H2BC correlation of H-1′/C-2′, giving the relative structure of **4** shown in Figure 1. Although 1D NMR and HRESIMS data for probreynin I (**4**) were reported in a Chinese patent in 2019 [13], no structural analysis based on 2D NMR spectroscopy has previously been described.

**Figure 6:**
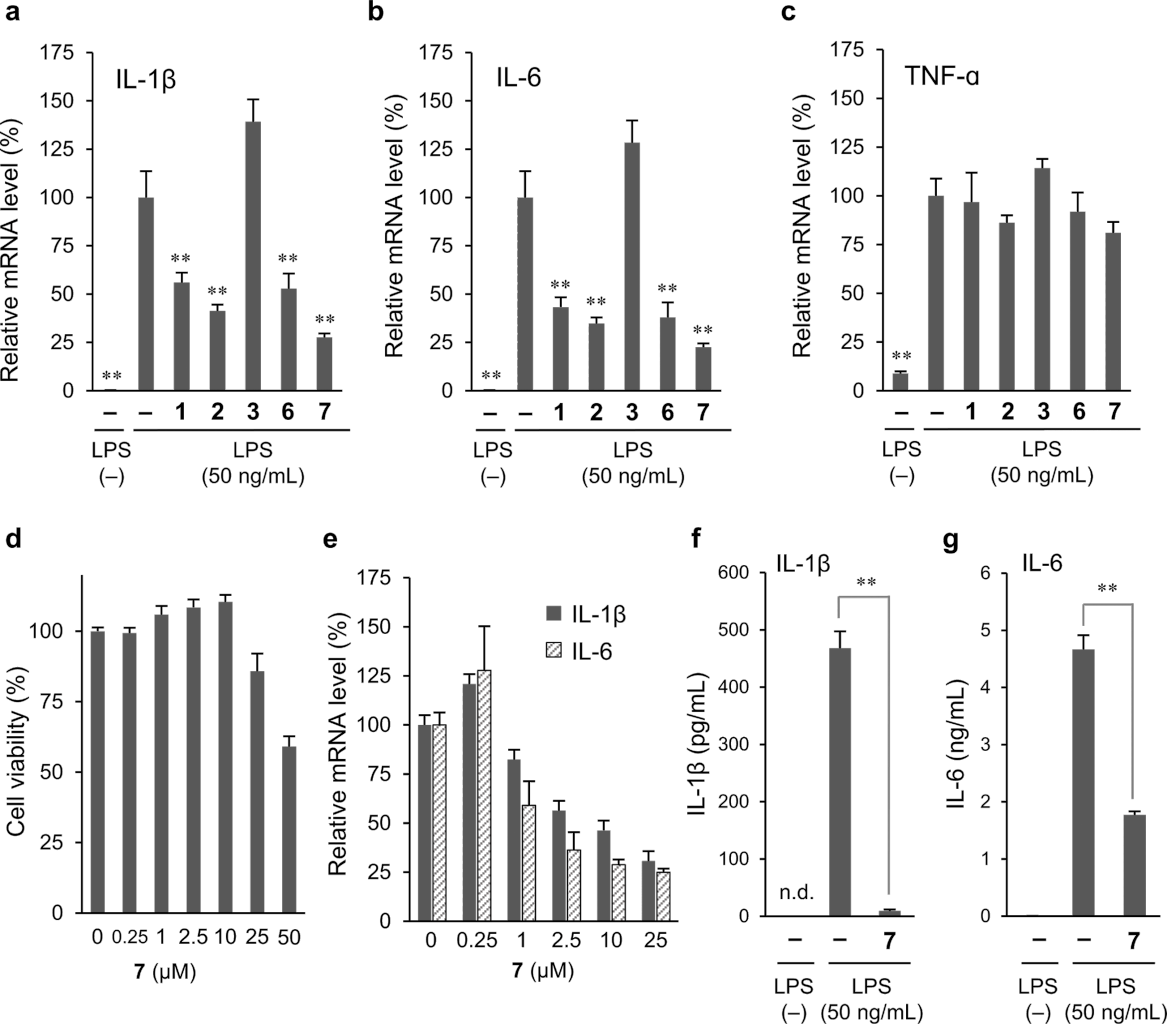
Anti-inflammatory effects of isolated sulfur-containing compounds. mRNA levels of a) IL-1β, b) IL-6, and c) TNF-α in RAW 264.7 cells preincubated with compounds **1**–**3**, **6**, or **7** (10 μM each) for 24 h, followed by treatment with LPS (50 ng/mL) for 2 h [significance was determined using one-way ANOVA followed by Dunnett’s test (**P < 0.01 versus the LPS-only group)]; d) concentration-dependent cytotoxicity of **7** in RAW 264.7 cells (cell viability was detected by an MTT-based colorimetric assay); e) mRNA levels of IL-1β and IL-6 in RAW 264.7 cells preincubated with different concentrations of compound **7** for 24 h, followed by treatment with LPS (50 ng/mL) for 2 h; levels of f) IL-1β and g) IL-6 in RAW 264.7 cell culture supernatants preincubated with compound **7** (10 μM) for 24 h, followed by treatment with LPS (50 ng/mL) for 12 h [significance was determined using an unpaired two-tailed Student’s *t* test (**P < 0.01); n.d. indicates not detected, i.e., below the detection limit].

He et al. reported that epibreynin D, which bears the same aglycone moiety as compounds **2** and **7**, exhibits anti-inflammatory effects in mice and rat models [12]. To investigate the anti-inflammatory effects of the sulfur-containing compounds isolated in this study, we investigated the lipopolysaccharide (LPS)-induced production of proinflammatory cytokines – interleukin (IL)-1β, IL-6, and TNF-α – in RAW 264.7 murine macrophage cells by quantitative real-time polymerase chain reaction (qRT-PCR) analysis. The sulfur-containing spiroketals **1**–**3**, **6**, and **7** were tested, whereas compound **4** was not because of its lower yield. The LPS-induced increases in IL-1β and IL-6 mRNA levels were significantly inhibited by compounds **1**, **2**, **6**, and **7** but enhanced by compound **3** (Figure 6a and 6b). In addition, the LPS-induced increase in TNF-α mRNA levels was not affected by any of the tested compounds (Figure 6c).

Further investigations of anti-inflammatory activity were focused on compound **7**, which showed the strongest inhibitory effect, although not significantly. The 3-(4,5-dimethylthiazol-2-yl)-2,5-diphenyltetrazolium bromide (MTT) assay revealed that compound **7** was cytotoxic to RAW 264.7 cells at concentrations above 25 μM (Figure 6d). Furthermore, **7** suppressed the LPS-induced increases in IL-1β and IL-6 transcriptional levels in a concentration-dependent manner from 1 or 2.5 μM (Figure 6e). Thus, while cytotoxicity was likely involved in the suppression of mRNA levels at high concentrations (>25 μM) of compound **7**, anti-inflammatory effects occurred at lower concentrations without cytotoxicity. The production of IL-1β and IL-6 was also evaluated by an enzyme-linked immunosorbent assay (ELISA) using culture supernatants. The LPS-stimulated increase in IL-1β and IL-6 production was suppressed by compound **7** at a concentration of 10 μM (Figure 6f and 6g). Thus, compound **7** inhibited LPS-stimulated IL-1β and IL-6 mRNA and protein expression in RAW 264.7 cells. Although confirmed only at the mRNA level, **1**, **2**, and **6** likely exhibit similar behavior, suggesting that the steric configuration of the sulfoxide group and the structure of the sugar chain may not influence the anti-inflammatory effect. In contrast, compound **3** did not show anti-inflammatory activity, suggesting that a thiophene ring or a sulfoxide group in breynins is involved in the onset of anti-inflammatory action.

## Conclusion

The biosynthesis of breynins with novel skeletons remains poorly understood, especially with respect to the sulfur source. In this study, we isolated a new aglycone, probreynogenin (**3**), together with a known sesquiterpenoid, phyllaemblic acid (**5**), suggesting that breynin formation may involve the attachment of a mercaptoacetamide to **5**, followed by cyclization. However, to the best of our knowledge, the origin of mercaptoacetamide has not been reported and further biosynthetic exploration is needed. We also isolated two new sulfur-containing spiroketals (**1** and **2**) with a tetrasaccharide moiety. For terpenoid glycosides, the ^1^H NMR spectrum becomes more complex as the number of sugar residues and their lengths increase, which complicates 2D NMR analysis. In such cases, commonly used TOCSY measurements can be supplemented by H2BC and HSQC-TOCSY correlations to analyze C–C linkages. Further studies are underway to investigate the mechanism of inflammation suppression by breynins and epibreynins.

## Experimental

### General experimental procedures

Optical rotation values were determined using a JASCO P-1020 polarimeter. UV spectra were recorded using a Hitachi U-2900 spectrometer. ECD spectra were acquired with a JASCO J-820 spectropolarimeter and IR spectra were recorded using a Shimadzu FTIR-8400S spectrophotometer. NMR spectra were acquired with a JEOL JNM-ECZ 400S spectrometer with tetramethylsilane as an internal standard. ESI–MS data were obtained using an Agilent 6230 LC/TOF mass spectrometer. HPLC was performed on a Hitachi HPLC system equipped with an L-2130 pump, an L-2200 autosampler, an L-2300 column oven, and an L-2455 diode array detector. Silica gel AP-300 (Toyota Kako), Sephadex LH-20 (GE Healthcare), and Cosmosil 75C_18_-OPN (Nacalai Tesque) were used for column chromatography (CC). Silica gel 60 F_254_ and RP-18 F_254S_ (Merck) were used for TLC analysis.

### Plant material

*B. disticha* was cultivated in the greenhouse of Setsunan University medicinal plant garden (Osaka, Japan) in September 2014. Taxonomic identification was performed by an expert botanist, Ms. Hiroko Murata (Setsunan University, retired). A voucher specimen (GPU-0812) has been deposited at the Gifu Pharmaceutical University for future reference.

### Extraction and isolation

The dried roots (767 g) of *B. disticha* were extracted with MeOH (5 L × 3 times) at room temperature, and the solution was evaporated in vacuo to afford a MeOH extract (47.2 g). The MeOH extract was partitioned three times with 1.6 L of ethyl acetate and water 1:1 (v/v), and the water-soluble fraction was further partitioned three times with 800 mL of *n*-butanol. The ethyl acetate and *n*-butanol solutions were concentrated in vacuo to yield the ethyl acetate fraction (11.8 g) and *n*-butanol fraction (7.4 g), respectively. The ethyl acetate fraction was separated by silica gel CC with CHCl_3_/MeOH (stepwise gradient of 100:1, 50:1, 30:1, 20:1, 15:1, 10:1, 8:1, and 0:1, v/v) as the eluent. The fractions were pooled by TLC analysis to yield seven combined fractions (Frs. E1–E7). Frs. E5 and E6 were further separated on a Sephadex LH-20 column eluted with MeOH to obtain compounds **5** (15.2 mg) and **3** (13.3 mg), respectively. The *n*-butanol fraction was separated on a Sephadex LH-20 column eluted with MeOH. The fractions were pooled by TLC analysis to yield four combined fractions (Frs. B1–B4). Fr. B2 was further separated on an ODS column eluted with MeOH/H_2_O (stepwise gradient of 2:3, 1:1, 3:2, and 1:0, v/v) to yield four combined subfractions. The second subfraction was separated by silica gel CC with ethyl acetate/MeOH 2:1 (v/v) to obtain compounds **1** (95.8 mg) and **6** (109.7 mg). The third subfraction was subjected to silica gel CC with ethyl acetate/MeOH 2:1 (v/v) to yield compounds **2** (79.0 mg) and **7** (44.1 mg). The fourth subfraction was purified by silica gel CC with ethyl acetate/MeOH 10:1 (v/v) to obtain compound **4** (1.2 mg).

Breynin J (**1**): amorphous, colorless powder. [α]_D_^22^ −8.0 (*c* 0.05, MeOH); UV λ_max_ (MeOH) nm (log ε): 257 (4.09); IR (KBr) cm^−1^: 3414, 2969, 2936, 2888, 1782, 1695, 1609, 1516, 1456, 1395, 1348, 1314, 1279, 1167, 1117, 1078, 1036, 854, 831, 773, 741, 700, 667, 619, 550, 511, 471; ^1^H and ^13^C NMR data, see Table 1 and Table 2. HRESIMS (*m/z*): [M + Na]^+^ calcd for C_45_H_64_O_28_SNa, 1107.3197; found, 1107.3177 .

Epibreynin J (**2**): amorphous, colorless powder. [α]_D_^22^ −45.2 (*c* 0.05, MeOH); UV λ_max_ (MeOH) nm (log ε): 257 (4.08); IR (KBr) cm^−1^: 3404, 2969, 2936, 2888, 1734, 1694, 1609, 1516, 1346, 1314, 1279, 1167, 1119, 1078, 1038, 1005, 854, 831, 773, 698, 667, 619, 586, 548, 509, 471; ^1^H and ^13^C NMR data, see Table 1 and Table 2; HRESIMS (*m/z*): [M + Na]^+^ calcd for C_45_H_64_O_28_SNa, 1107.3197; found, 1107.3178 .

Probreynogenin (**3**): amorphous, colorless solid. [α]_D_^22^ +50.8 (*c* 0.05, MeOH); UV λ_max_ (MeOH) nm (log ε): 257 (4.10); IR (KBr) cm^–1^: 3430, 2969, 2936, 2889, 1780, 1674, 1609, 1593, 1514, 1437, 1391, 1360, 1344, 1312, 1281, 1167, 1117, 1084, 1060, 1044, 999, 980, 853, 773; ^1^H and ^13^C NMR data, see Table 1. HRESIMS (*m/z*): [M + Na]^+^ calcd for C_23_H_27_NO_11_SNa, 548.1197; found, 548.1197 .

Probreynin I (**4**): amorphous, colorless solid. [α]_D_^22^ +21.6 (*c* 0.05, MeOH); UV λ_max_ (MeOH) nm (log ε): 257 (3.65); IR (KBr) cm^–1^: 3404, 2965, 2936, 2888, 1780, 1749, 1670, 1609, 1514, 1456, 1387, 1373, 1362, 1341, 1314, 1279, 1167, 1117, 1078, 773, 617, 584, 550; ^1^H and ^13^C NMR data, see Table 1 and Table 2; HRESIMS (*m/z*): [M + Na]^+^ calcd for C_35_H_47_NO_21_SNa, 872.2253; found, 872.2241.

### Identification of sugars in **1** and **2**

Compound **1** (5 mg) was dissolved in 0.5 M hydrochloric acid in a glass centrifuge tube and heated at 95 °C for 2 h. The solution was neutralized with 0.5 M NaOH and concentrated in vacuo. To the residue was added a solution of ʟ-cysteine methyl ester hydrochloride (2 mg) in pyridine (200 μL), and the mixture was stirred at 65 °C for 1 h. Then, a solution of *o*-tolyl isothiocyanate (2.2 μL) in pyridine (200 μL) was added and the resulting mixture was stirred at 65 °C for 1 h. The final mixture was filtered and analyzed by HPLC without dilution. HPLC separation was performed on a 150 × 4.6 mm i.d. CAPCELL PAK C18 UG120 column (Osaka Soda) at 35 °C with isocratic elution using 20% CH_3_CN in 50 mM H_3_PO_4_ for 30 min and subsequent column washing with 90% CH_3_CN at a flow rate 0.8 mL/min. Detection at 250 nm was performed using a photodiode array detector. Compounds **2**, **6**, and **7** were derivatized and analyzed in a similar manner. Furthermore, derivatives of authentic sugars were analyzed, and peaks were observed at 8.34 (ᴅ-mannose), 12.17 (ᴅ-galactose), 12.74 (ʟ-glucose), 13.96 (ᴅ-glucose), 15.18 (ʟ-arabinose), 16.20 (ᴅ-arabinose), and 22.14 (ʟ-rhamnose) min.

### Computational method

Conformers of **1′**, **2′**, and **3** were generated using CONFLEX 8 with the MMFF94s force field [19] and a search limit of 5 kcal/mol, respectively. Similar conformers were excluded, yielding 9 conformers for **1′**, 10 conformers for **2′**, and 24 conformers for **3**. The geometry of each conformer was optimized using DFT calculations at the B3LYP/6-31G(d) level of theory [20–21] with the conductor-like polarizable continuum model (CPCM) solvent model (MeOH) and Gibbs free energy was calculated subsequent frequency calculations. Time-dependent (TD)-DFT calculations at the B3LYP/6-31+G(d,p) level with CPCM solvent model (MeOH) [22] were performed for the optimized conformers. The resulting ECD spectra calculated for each conformer were averaged using Boltzmann populations evaluated at 300 K from Gibbs free energy calculated from the frequency calculations.

### Cell culture

RAW 264.7 cells were maintained in Dulbecco’s modified Eagle’s medium (DMEM) supplemented with 10% fetal bovine serum (FBS), 50 U/mL penicillin, and 50 μg/mL streptomycin at 37 °C in a humidified 5% CO_2_ atmosphere. Test samples (compounds **1**–**3**, **6**, and **7**) were prepared to appropriate concentrations by dissolving in DMSO and diluting 1000-fold in medium. RAW 264.7 cells were preincubated with fresh medium containing test samples or vehicle (DMSO) for 24 h. After preincubation, LPS was added at a final concentration of 50 ng/mL.

### qRT-PCR analysis

Total RNA was extracted using RNAiso Plus (Takara Bio) after 2 h of LPS treatment. cDNA was obtained by reverse transcription from 0.3 µg of total RNA using the ReverTra Ace RT-qPCR Master Mix with gDNA remover (Toyobo). qRT-PCR analysis was performed using a StepOnePlus real-time PCR system (Thermo Fisher Scientific) with the Thunderbird SYBR qPCR Mix. The relative concentration of each sample was normalized to the β-actin mRNA level. The relative mRNA expression levels of the target genes were analyzed using the ΔΔCt method.

### MTT assay

To evaluate cell viability, RAW 264.7 cells were seeded into 96-well plates at a density of 5 × 10^4^ cells/well 24 hours before adding samples. The cells were treated with compound **7** at concentrations 0.25, 1, 2.5, 10, 25, and 50 µM for 24 h and then 0.2 mg/mL MTT solution was added after incubating for 3 h at 37 °C in a humidified 5% CO_2_ atmosphere, the culture supernatants were removed. The resulting dark blue crystals were dissolved in dimethyl sulfoxide, and the absorbance of the obtained solution was measured at 492 nm.

### ELISA

The concentrations of IL-1β and IL-6 in the culture medium after 12 h of LPS treatment were measured using Quantikine ELISA kits for mouse IL-1β and IL-6 (R&D Systems) according to the manufacturer’s instructions.

### Statistical analysis

All experiments were performed in triplicate or quadruplicate. Statistical analyses were performed using GraphPad Prism, version 6.0 and data are presented as mean ± standard deviation. An unpaired two-tailed Student’s *t*-test was used for comparisons between two groups. Dunnett’s test was used for multiple comparisons. *P* < 0.05 was considered statistically significant.

## Supporting Information

File 1 1D and 2D NMR data for compounds **1**–**4**.

File 2 Calculated energies and cartesian coordinates for the optimized conformers for **1′**, **2′**, and **3**.
