# Supplementary material for: Sulfur-containing spiroketals from Breynia disticha and evaluations of their anti-inflammatory effect
Source: Beilstein J Org Chem. 2023 Oct 19;19:1604–14. doi: 10.3762/bjoc.19.117 (PMC10616701; doi:10.3762/bjoc.19.117)
Supplement: File 1 — 1D and 2D NMR data for compounds 1–4. [file Beilstein_J_Org_Chem-19-1604-s001.pdf]

BEILSTEIN JOURNAL OF ORGANIC CHEMISTRY

Supporting Information

for

Sulfur-containing spiroketals from *Breynia disticha* and evaluations of their anti-inflammatory effect

Ken-ichi Nakashima, Naohito Abe, Masayoshi Oyama, Hiroko Murata and Makoto Inoue

Beilstein J. Org. Chem. **2023,** *19,* 1604–1614. doi:10.3762/bjoc.19.117

1D and 2D NMR data for compounds 1–4

License and Terms: This is a supporting information file under the terms of the Creative Commons Attribution License (https://creativecommons.org/licenses/by/4.0). Please note that the reuse, redistribution and reproduction in particular requires that the author(s) and source are credited and that individual graphics may be subject to special legal provisions.
The license is subject to the *Beilstein Journal of Organic Chemistry* terms and conditions: (https://www.beilstein-journals.org/bjoc/terms)

Table of contents

**Figure S1**	^{1}H NMR spectrum of **1** in CD_3OD	S3
**Figure S2**	^{13}C NMR and DEPT135 spectra of **1** in CD_3OD	S4
**Figure S3**	1D-TOCSY spectra of **1** in CD_3OD	S5
**Figure S4**	DQF-COSY spectrum of **1** in CD_3OD	S6
**Figure S5**	HSQC spectrum of **1** in CD_3OD	S7
**Figure S6**	HMBC spectrum of **1** in CD_3OD	S8
**Figure S7**	HSQC-TOCSY spectrum of **1** in CD_3OD	S9
**Figure S8**	H2BC spectrum of **1** in CD_3OD	S10
**Figure S9**	2D ROESY spectrum of **1** in CD_3OD	S11
**Figure S10**	^{1}H NMR spectrum of **2** in CD_3OD	S12
**Figure S11**	^{13}C NMR and DEPT135 spectra of **2** in CD_3OD	S13
**Figure S12**	1D-TOCSY spectra of **2** in CD_3OD	S14
**Figure S13**	DQF-COSY spectrum of **2** in CD_3OD	S15
**Figure S14**	HSQC spectrum of **2** in CD_3OD	S16
**Figure S15**	HMBC spectrum of **2** in CD_3OD	S17
**Figure S16**	HSQC-TOCSY spectrum of **2** in CD_3OD	S18
**Figure S17**	H2BC spectrum of **2** in CD_3OD	S19
**Figure S18**	2D ROESY spectrum of **2** in CD_3OD	S20
**Figure S19**	^{1}H NMR spectrum of **3** in CD_3OD	S21
**Figure S20**	^{13}C NMR and DEPT135 spectra of **3** in CD_3OD	S22
**Figure S21**	DQF-COSY spectrum of **3** in CD_3OD	S23
**Figure S22**	HSQC spectrum of **3** in CD_3OD	S24
**Figure S23**	HMBC spectrum of **3** in CD_3OD	S25

**Figure S24**	NOESY spectrum of **3** in CD_3OD	S26
**Figure S25**	^1H NMR spectrum of **4** in CD_3OD	S27
**Figure S26**	^{13}C NMR and DEPT135 spectra of **4** in CD_3OD	S28
**Figure S27**	1D-TOCSY spectra of **4** in CD_3OD	S29
**Figure S28**	DQF-COSY spectrum of **4** in CD_3OD	S30
**Figure S29**	HSQC spectrum of **4** in CD_3OD	S31
**Figure S30**	HMBC spectrum of **3** in CD_3OD	S32
**Figure S31**	H2BC spectrum of **3** in CD_3OD	S33
**Figure S32**	^1H NMR spectrum of **6** in CD_3OD	S34
**Figure S33**	^{13}C NMR spectrum of **6** in CD_3OD	S35
**Figure S34**	^1H NMR spectrum of **7** in CD_3OD	S36
**Figure S35**	^{13}C NMR spectrum of **7** in CD_3OD	S37
**Figure S36**	HPLC chromatograms for sugar identification	S38

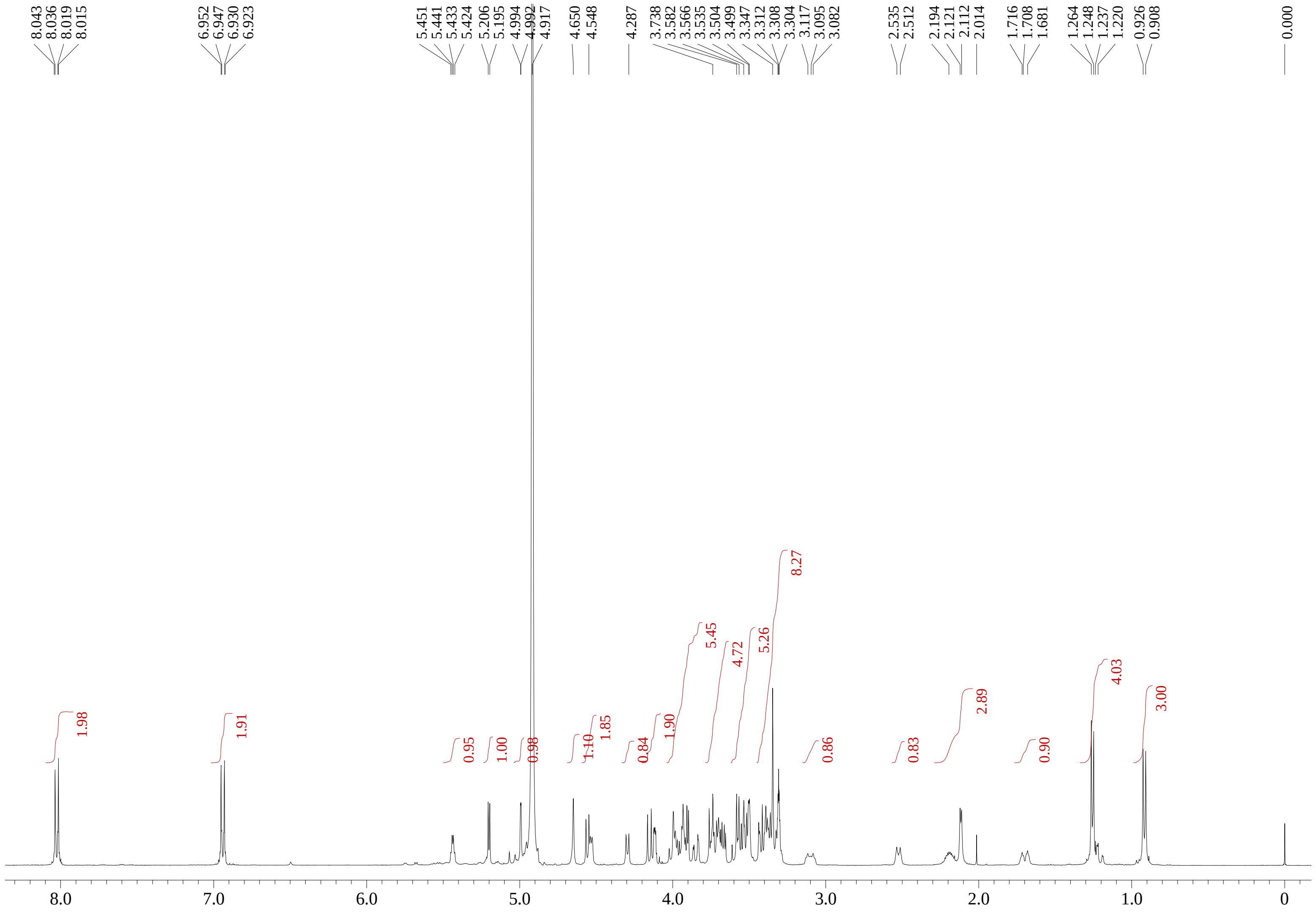

Figure S1 ^{1}H NMR spectrum of **1** in CD_3OD

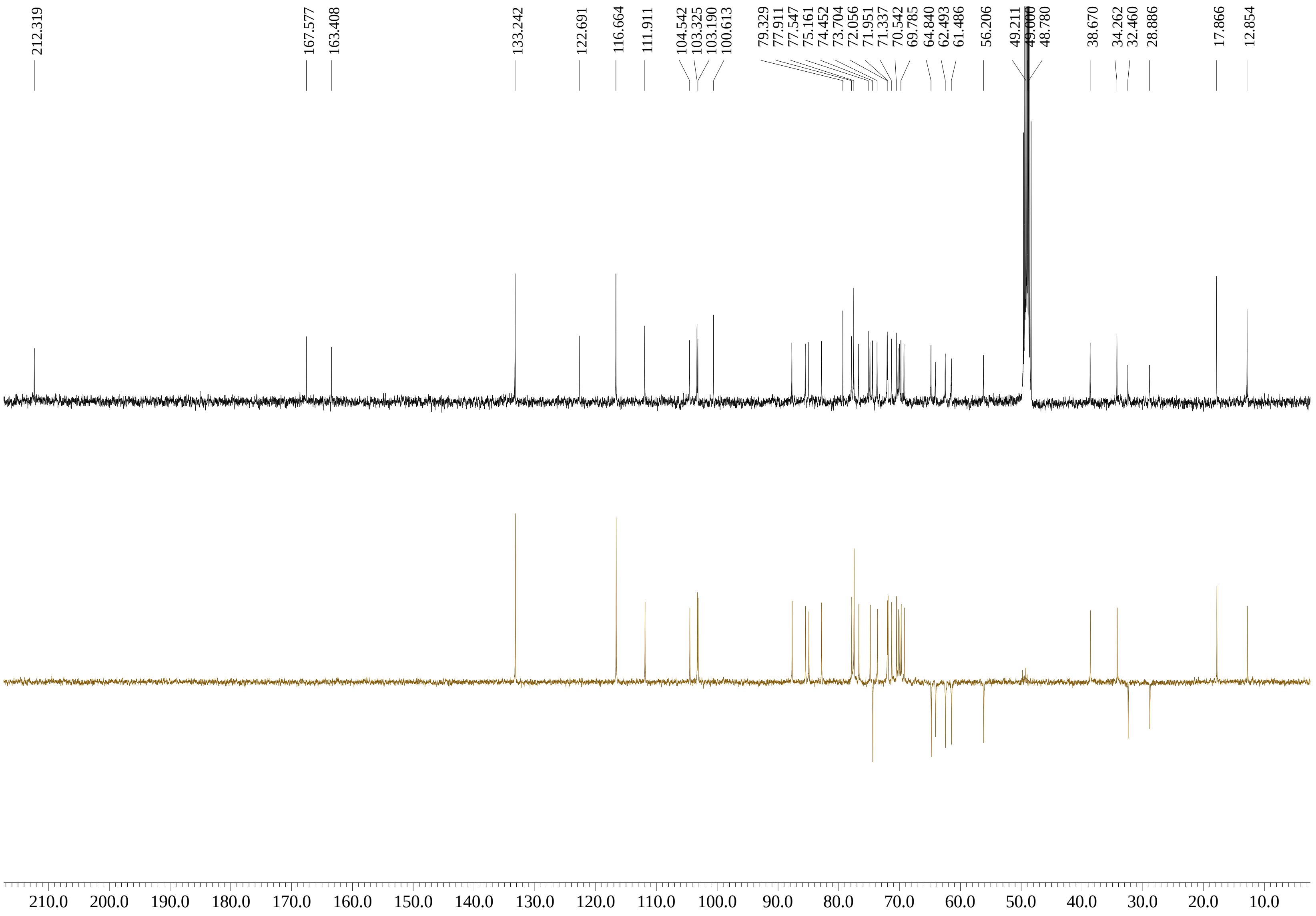

Figure S2 ^{13}C NMR and DEPT135 spectra of **1** in CD_3OD

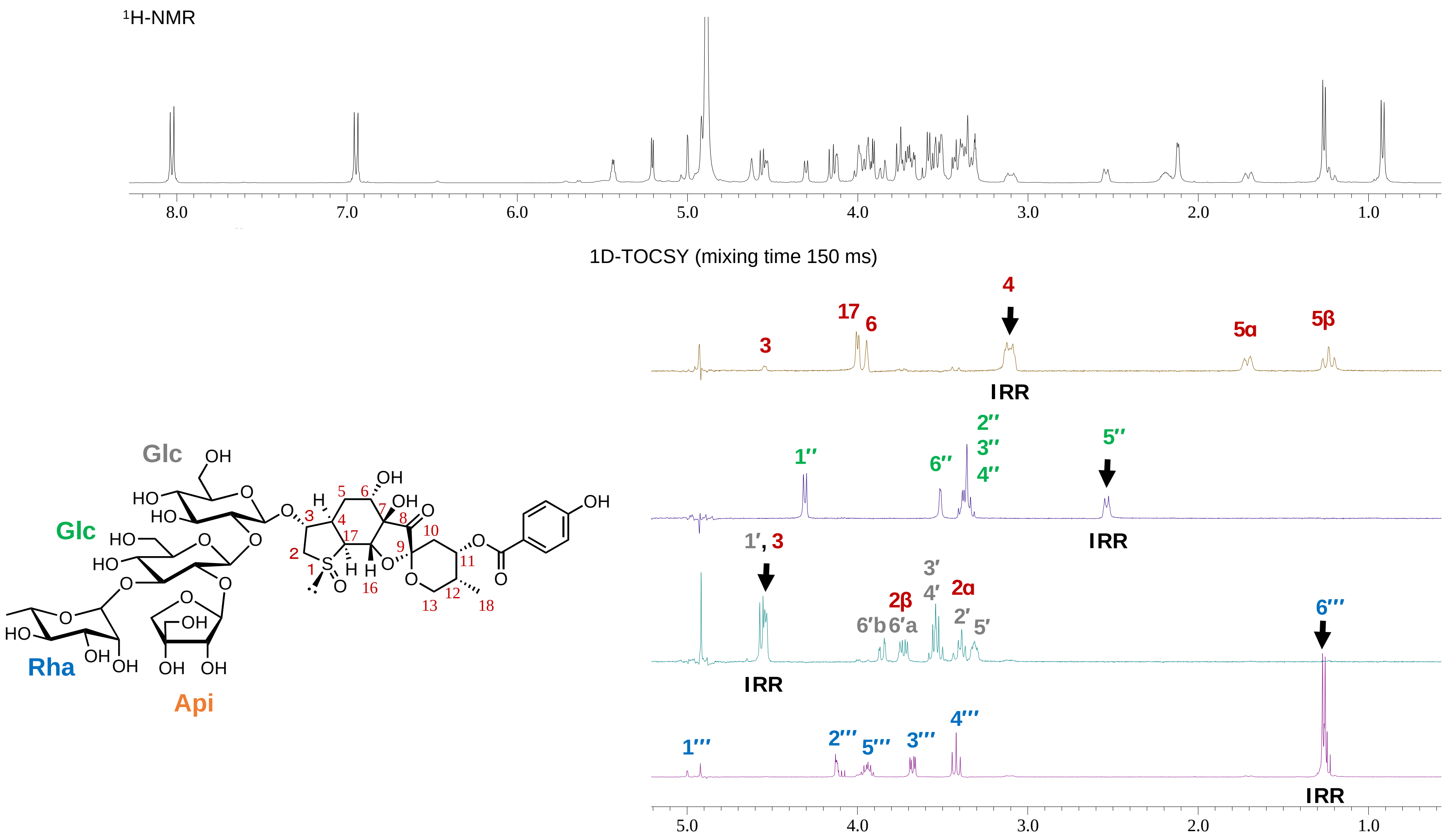

Figure S3 1D-TOCSY spectra of **1** in CD_3OD

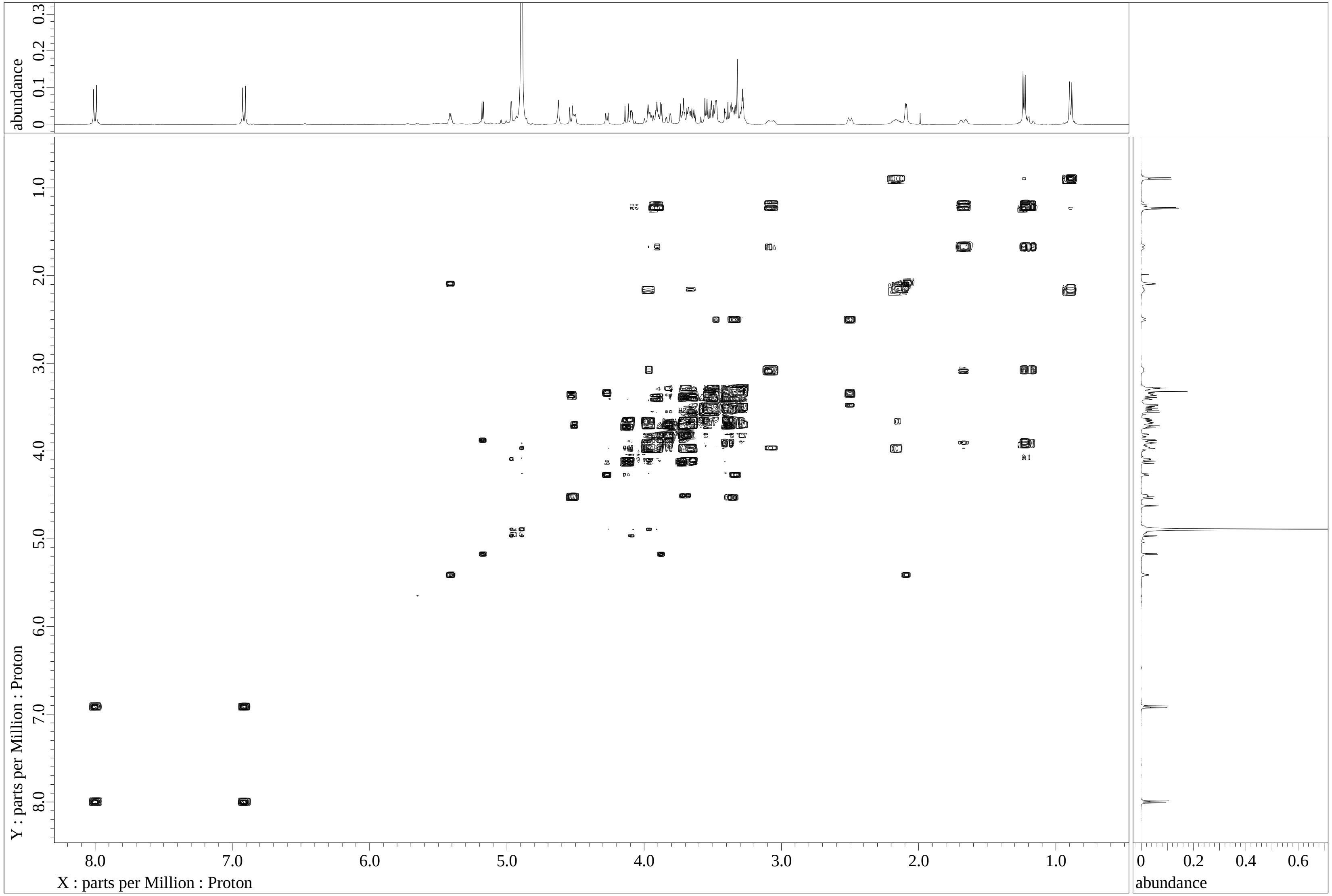

Figure S4 DQF-COSY spectrum of **1** in CD_3OD

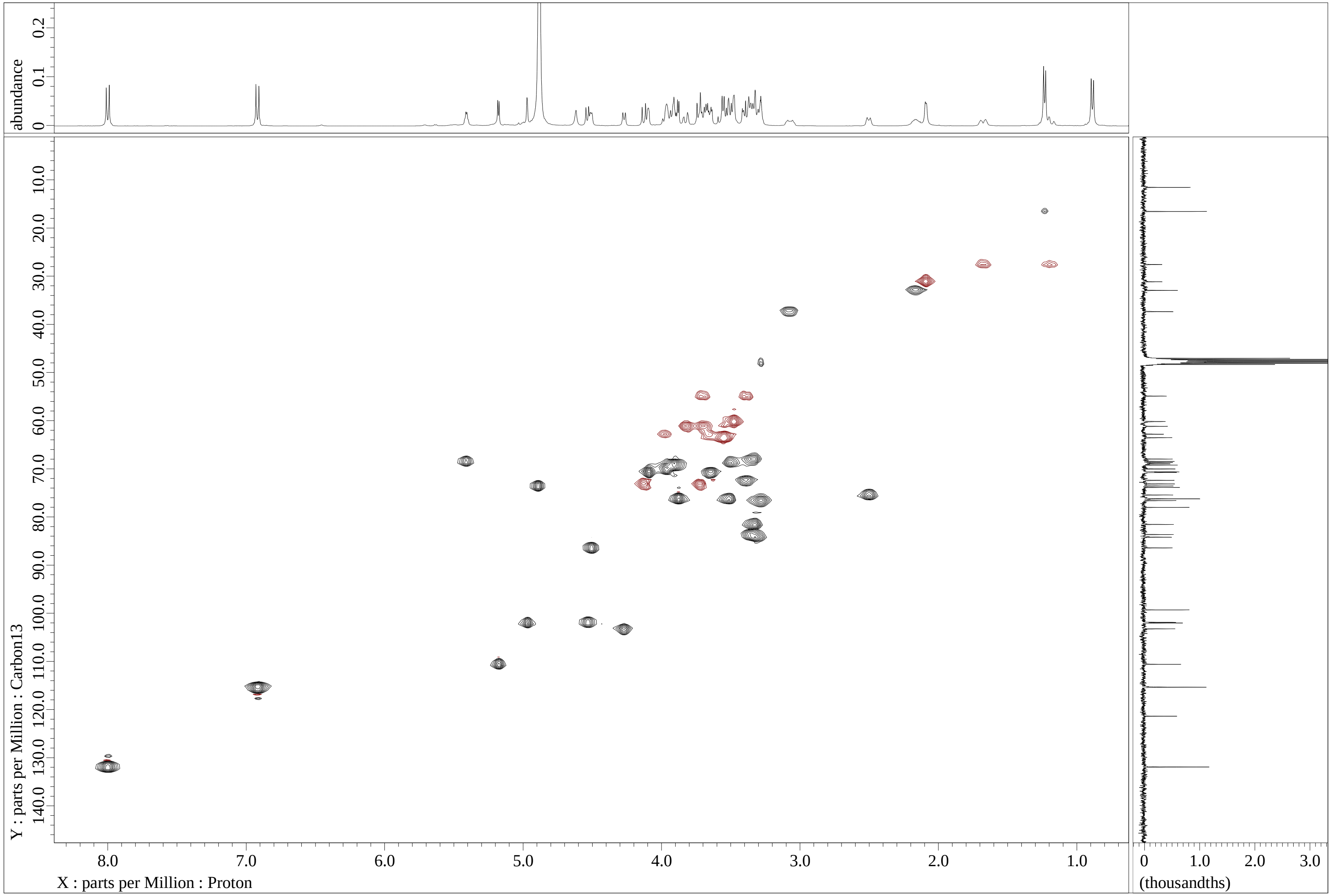

Figure S5 HSQC spectrum of **1** in CD_3OD

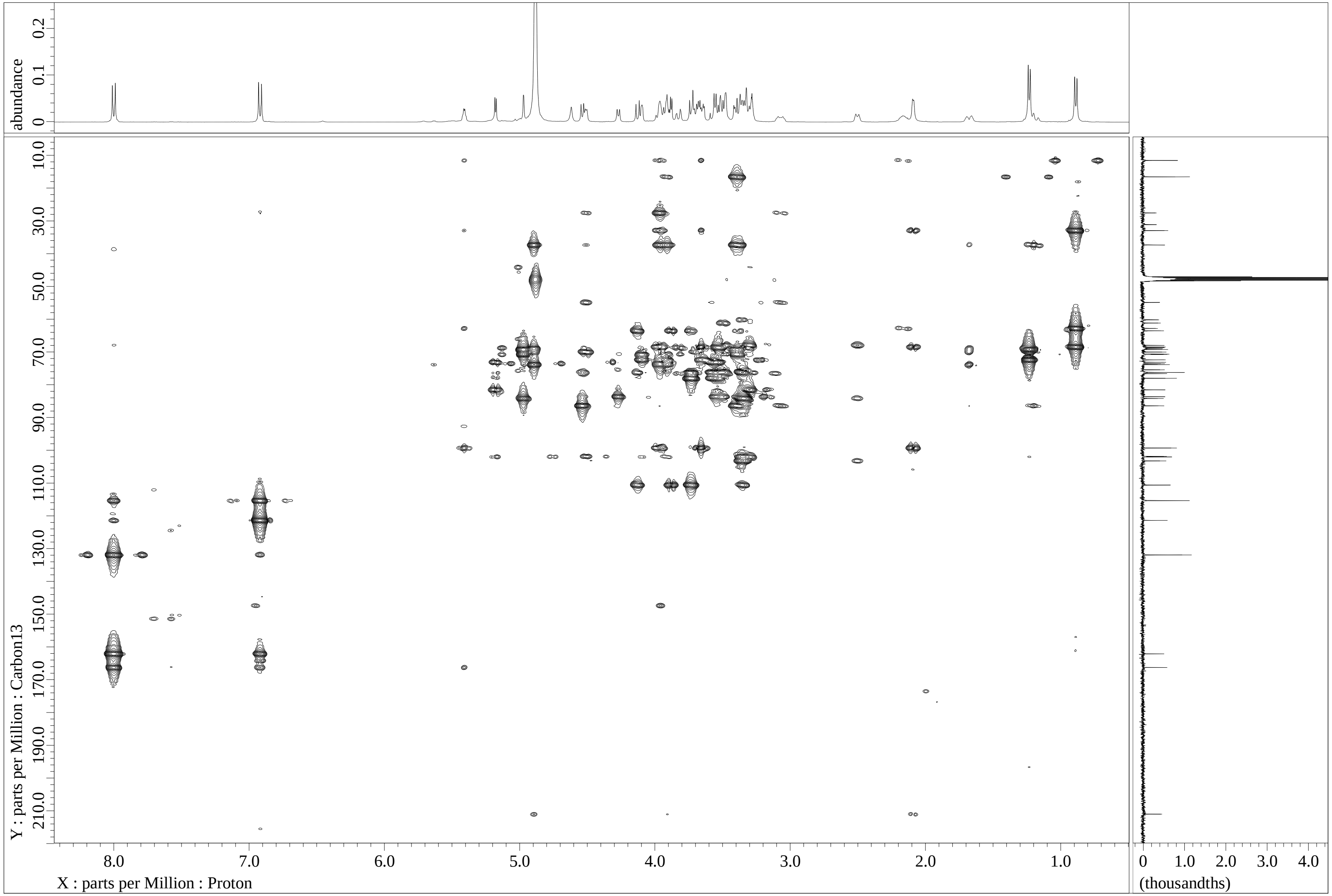

Figure S6 HMBC spectrum of **1** in CD_3OD

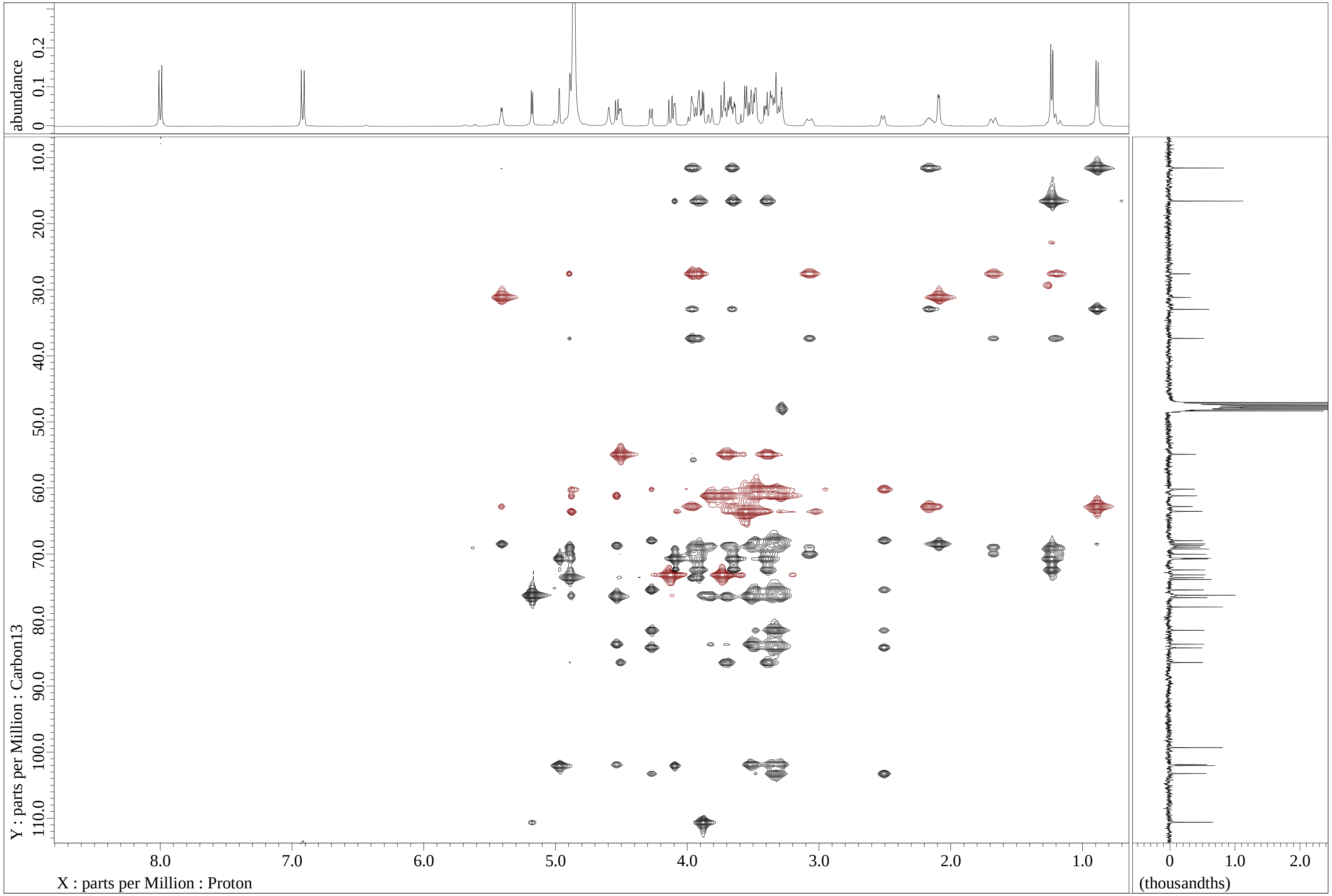

Figure S7 HSQC-TOCSY spectrum of **1** in CD_3OD

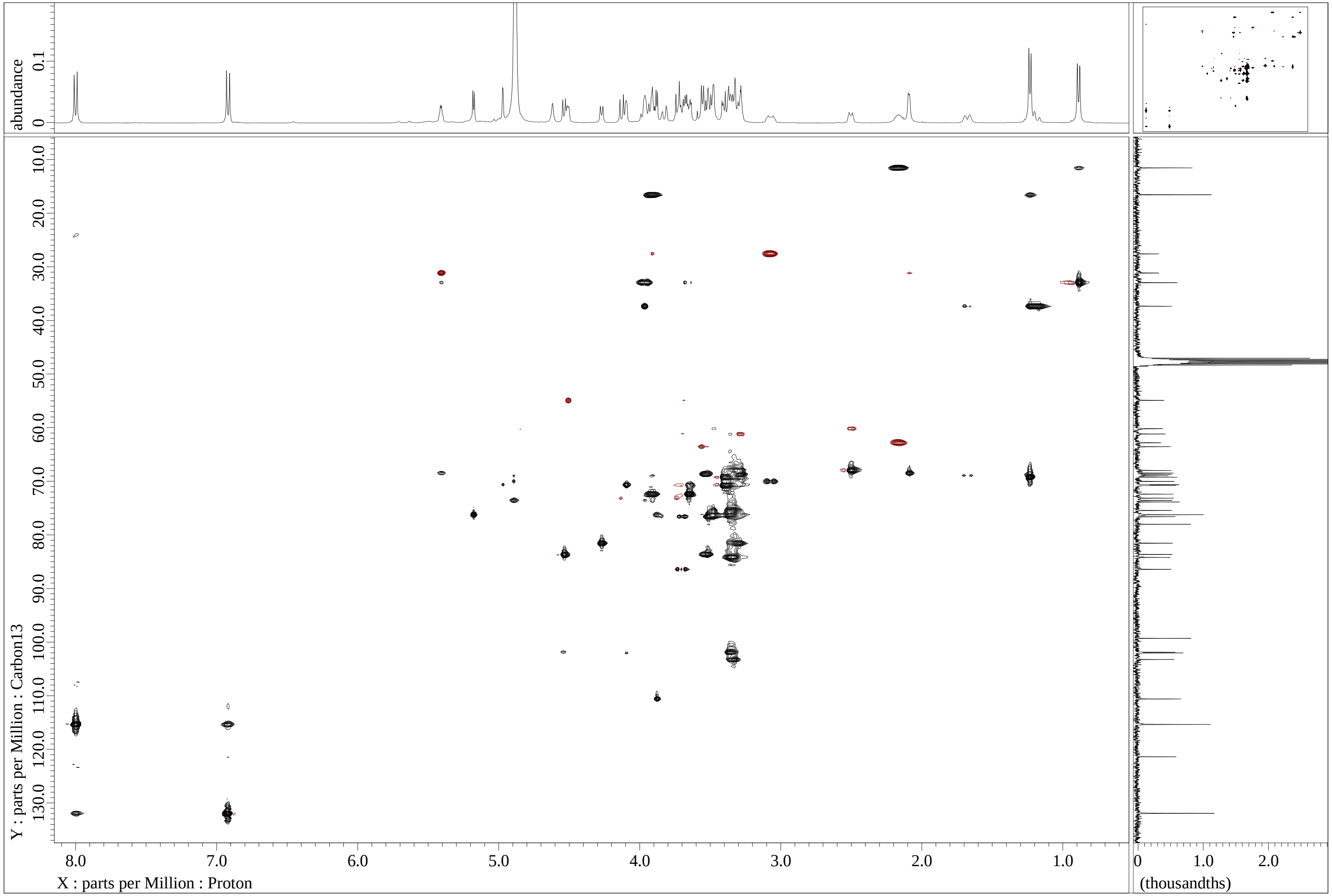

Figure S8 H2BC spectrum of **1** in CD_3OD

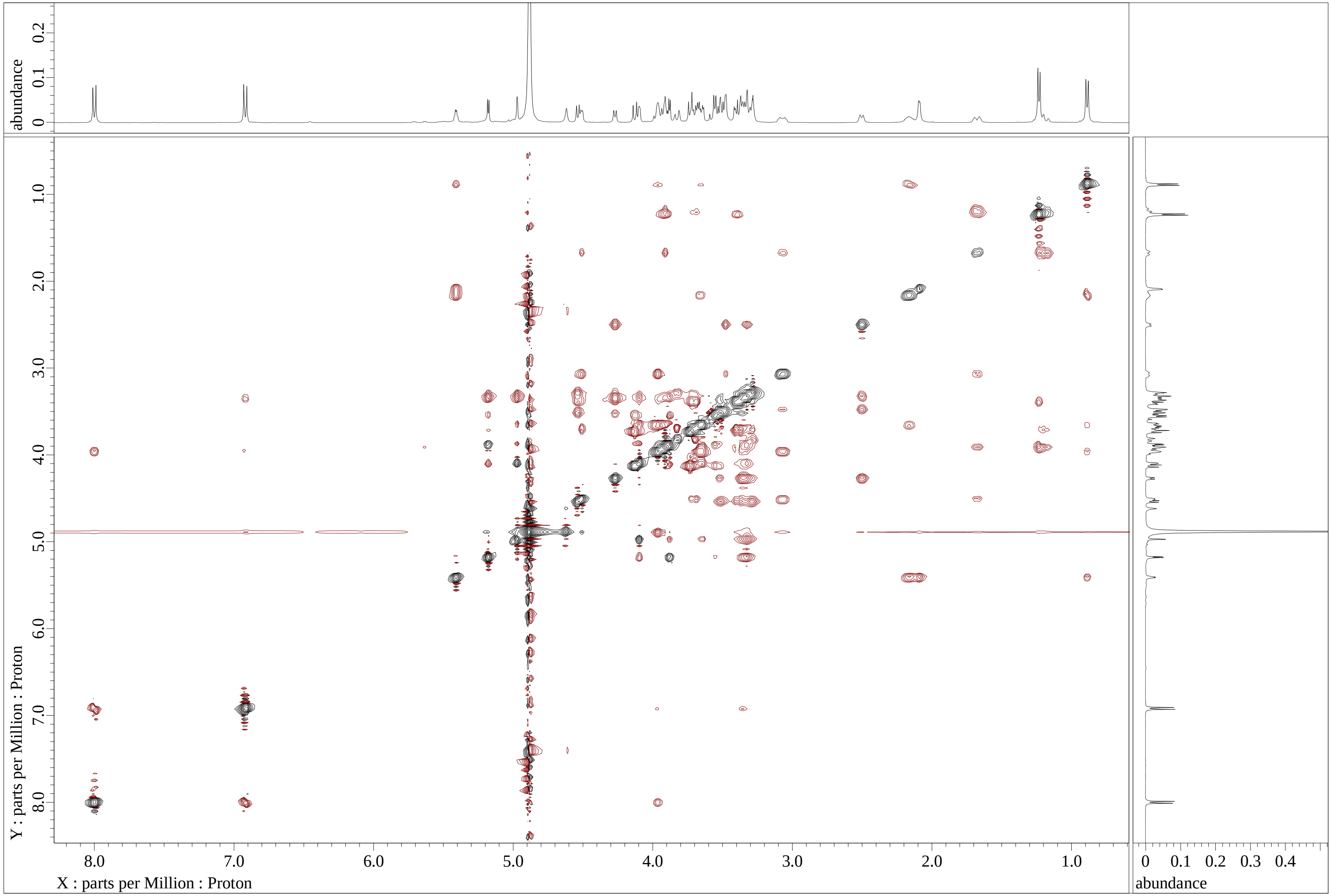

Figure S9 2D ROESY spectrum of **1** in CD_3OD

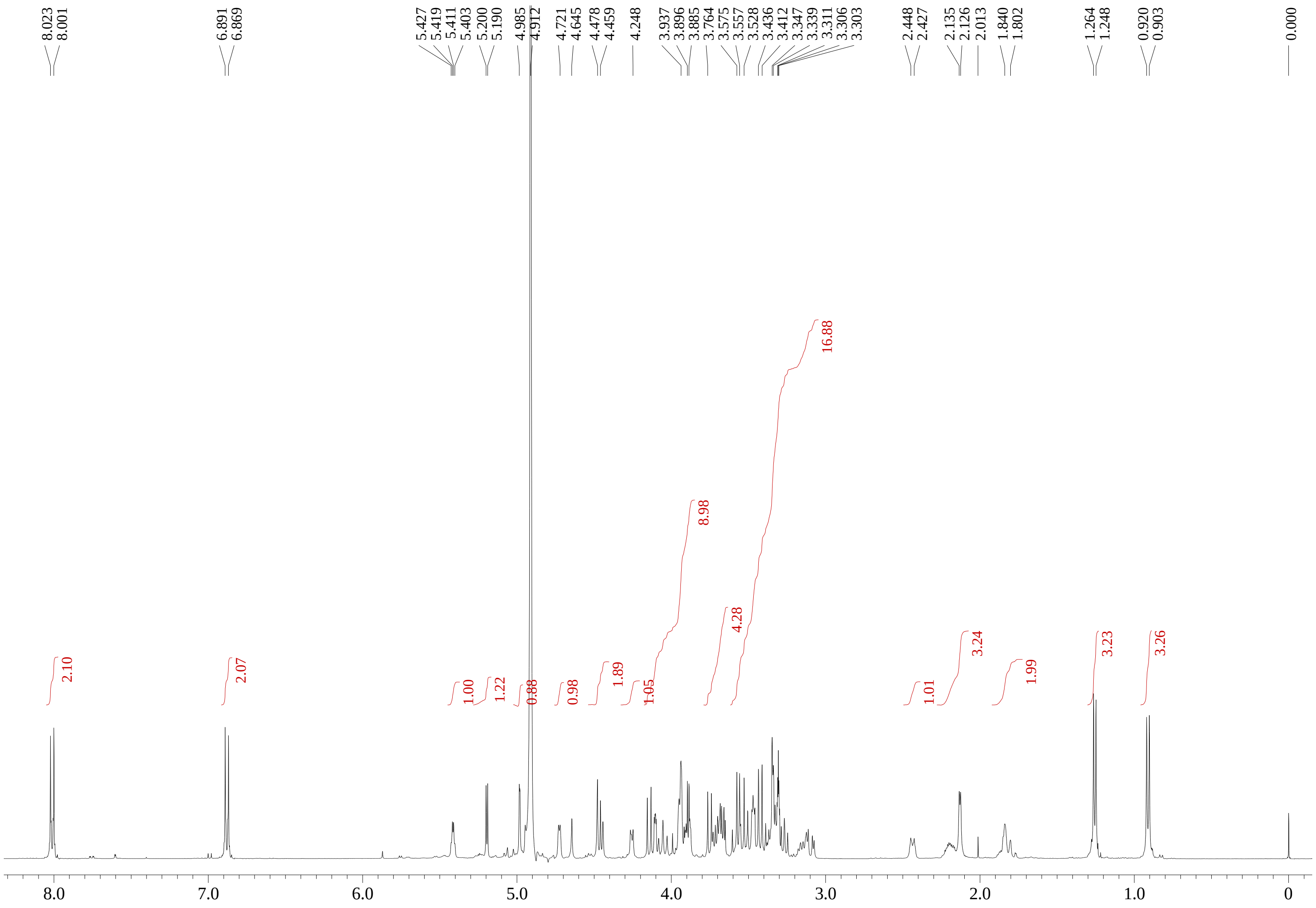

Figure S10 ^{1}H NMR spectrum of **2** in CD_3OD

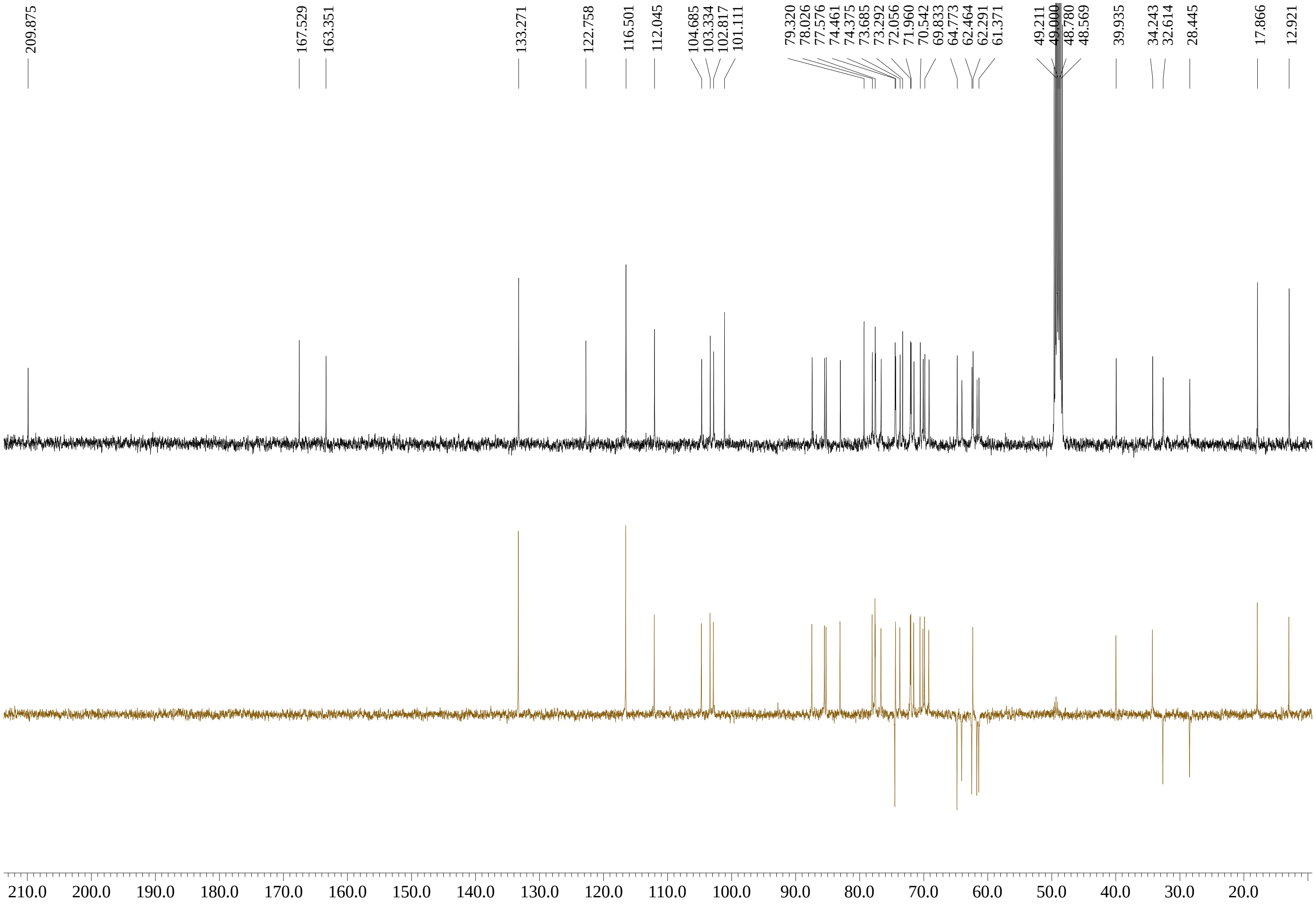

Figure S11 ^{13}C NMR and DEPT135 spectra of **2** in CD_3OD

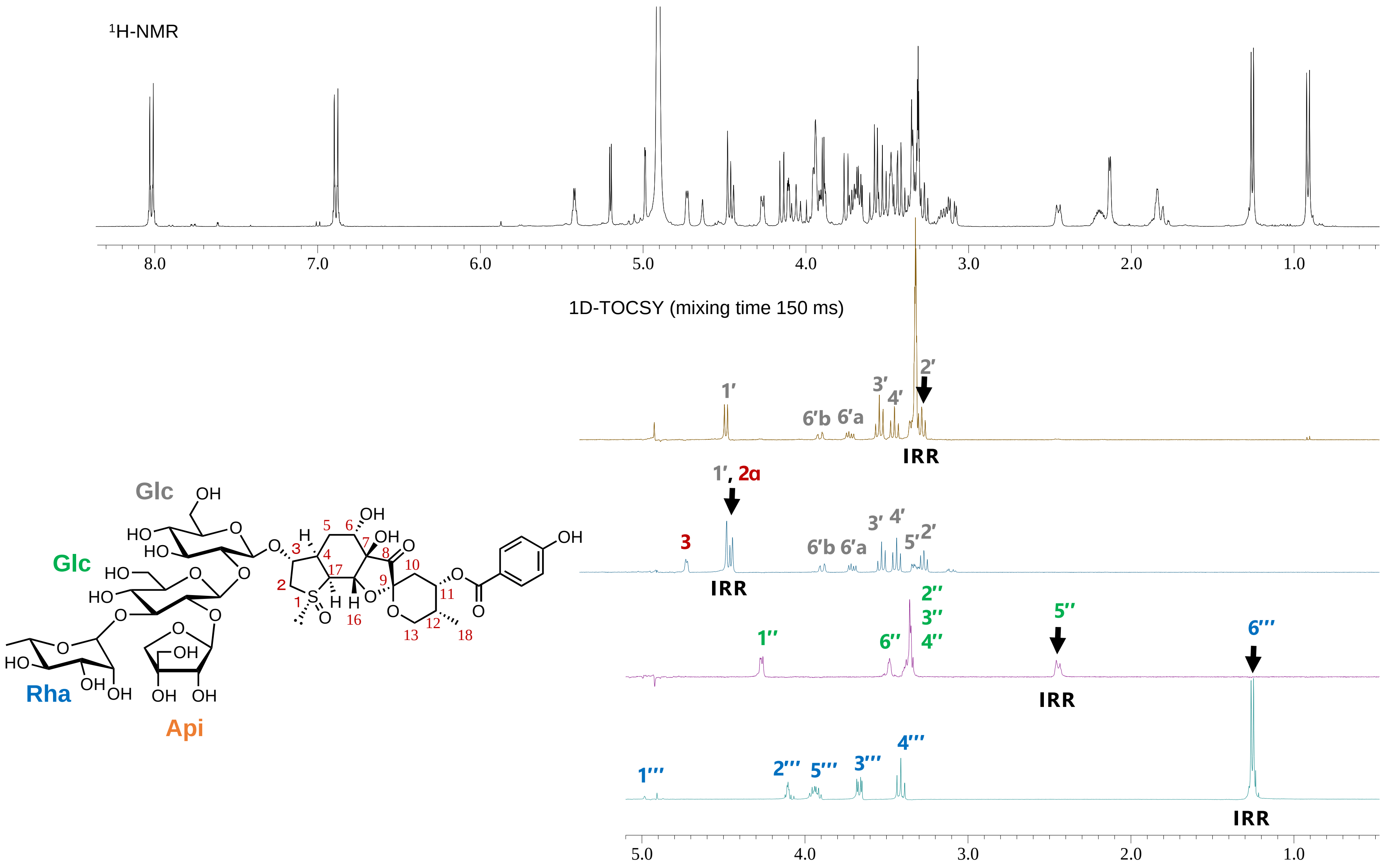

Figure S12 1D-TOCSY spectra of **2** in CD_3OD

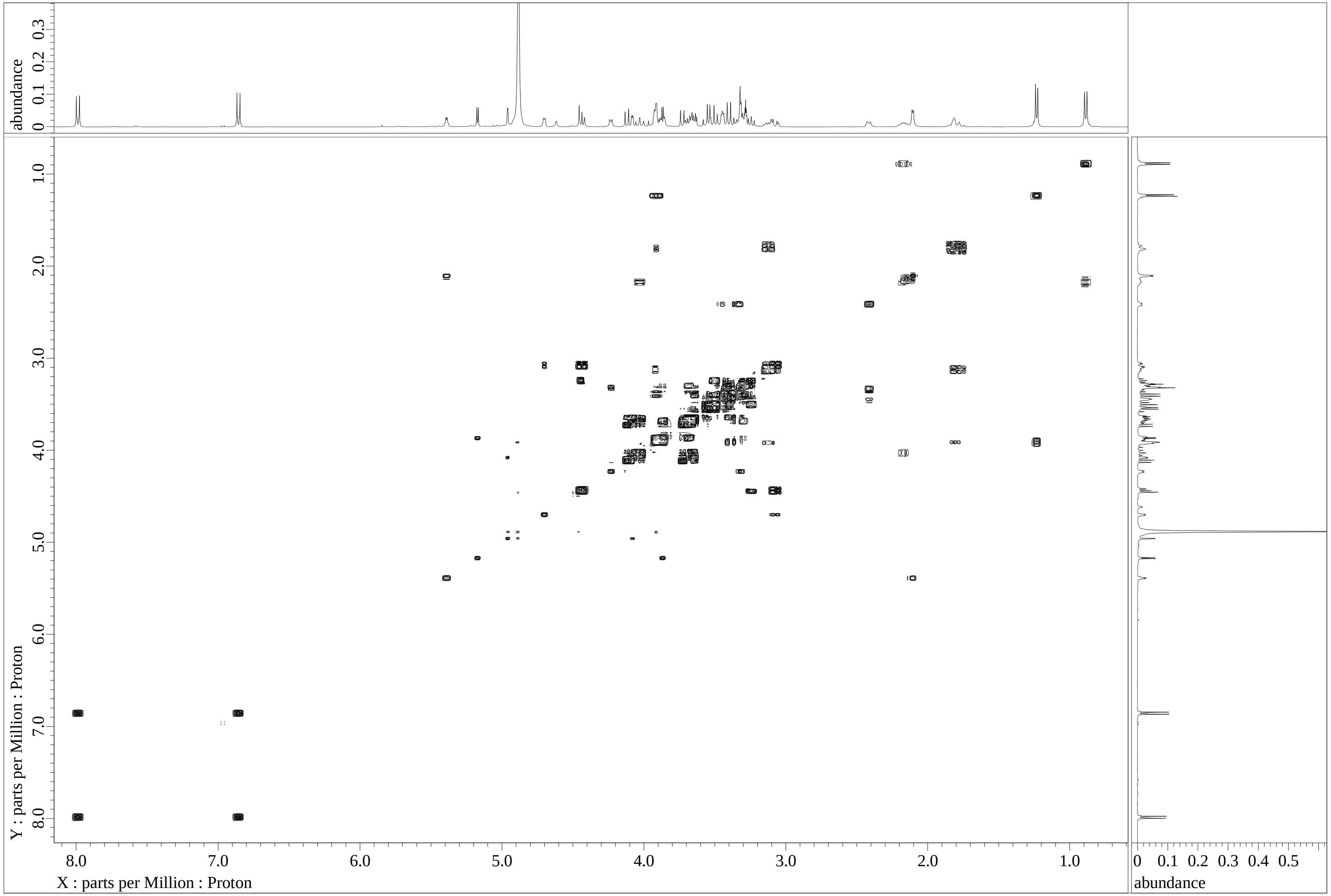

Figure S13 DQF-COSY spectrum of **2** in CD_3OD

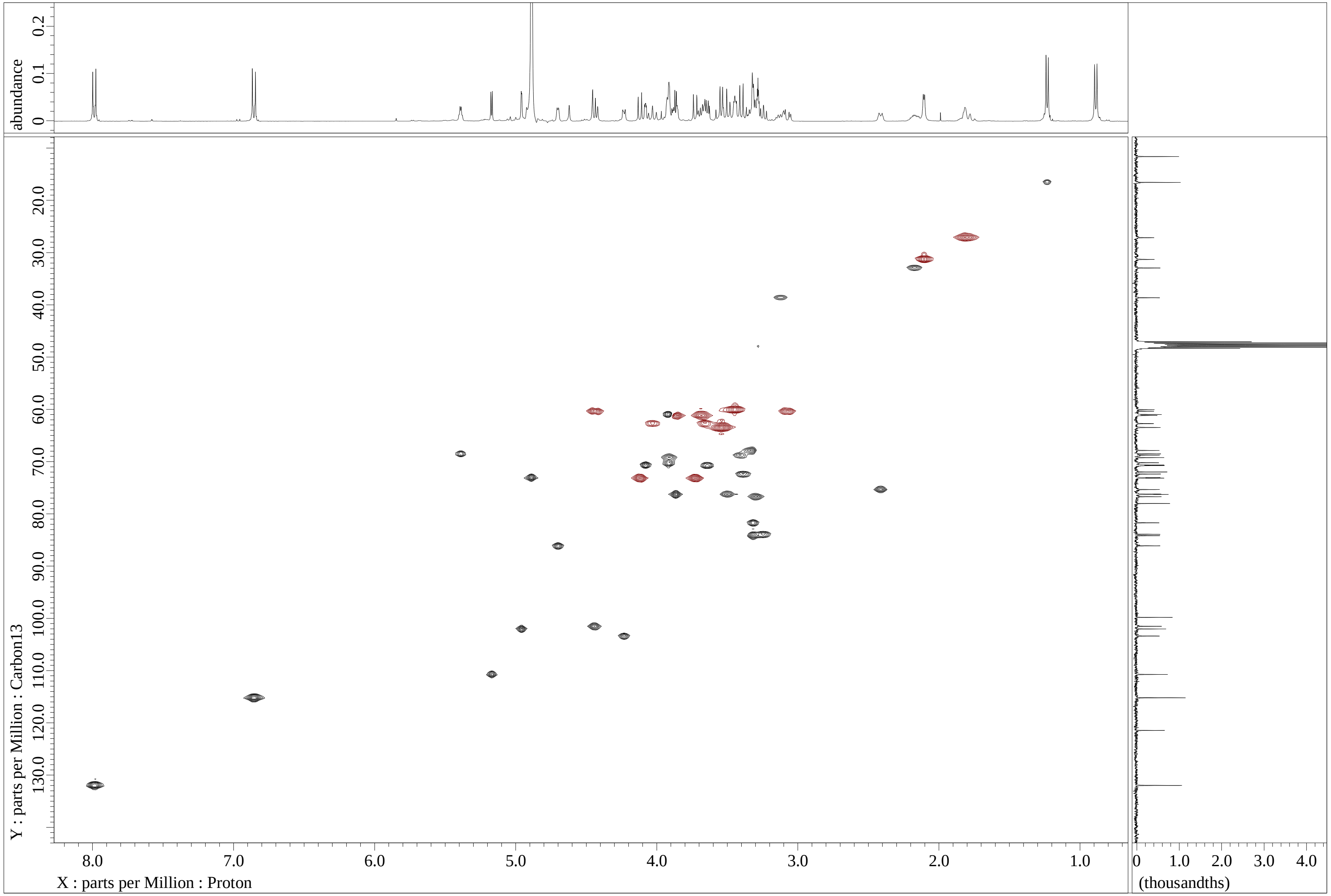

Figure S14 HSQC spectrum of **2** in CD_3OD

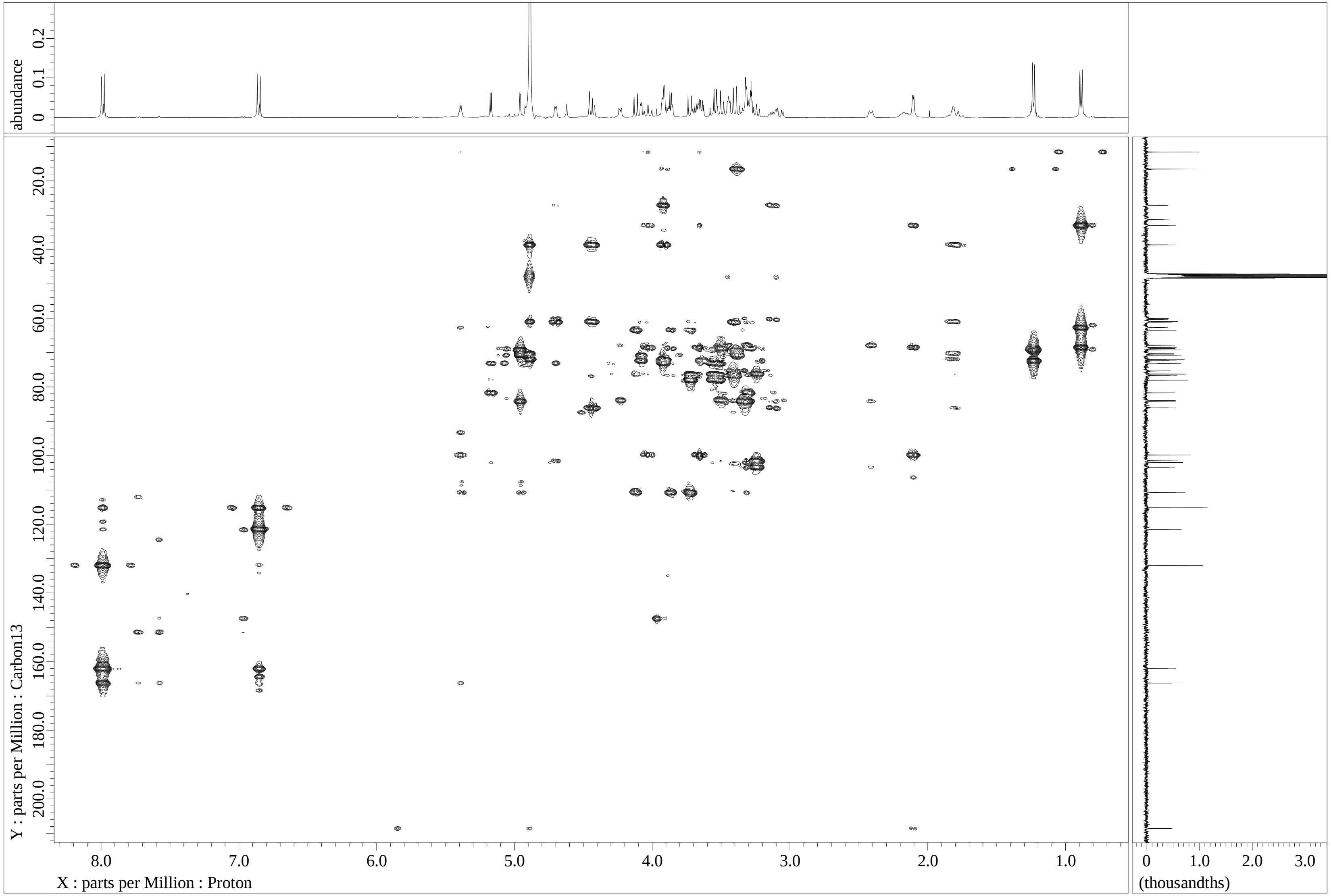

Figure S15 HMBC spectrum of **2** in CD_3OD

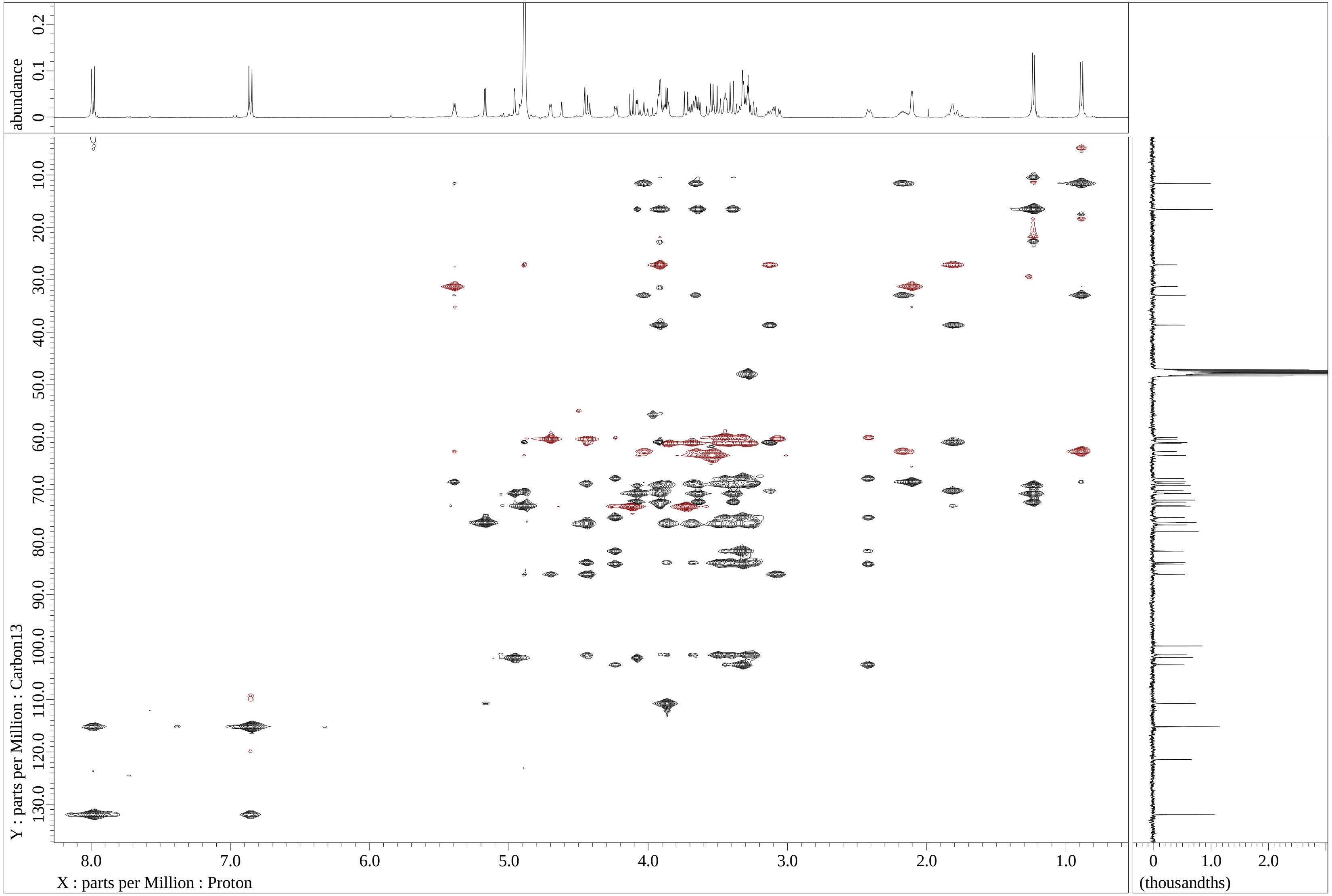

Figure S16 HSQC-TOCSY spectrum of **2** in CD_3OD

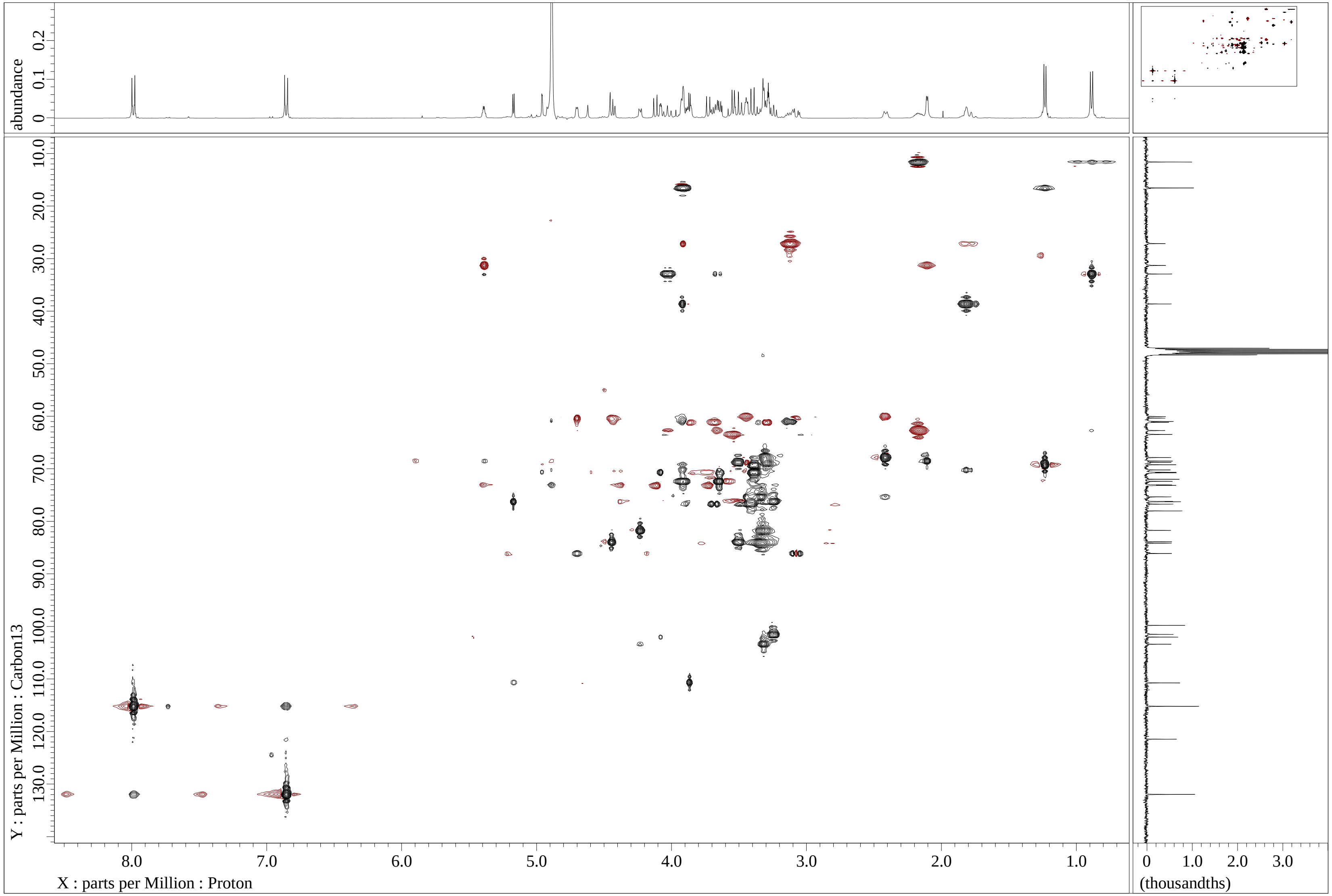

Figure S17 H2BC spectrum of **2** in CD_3OD

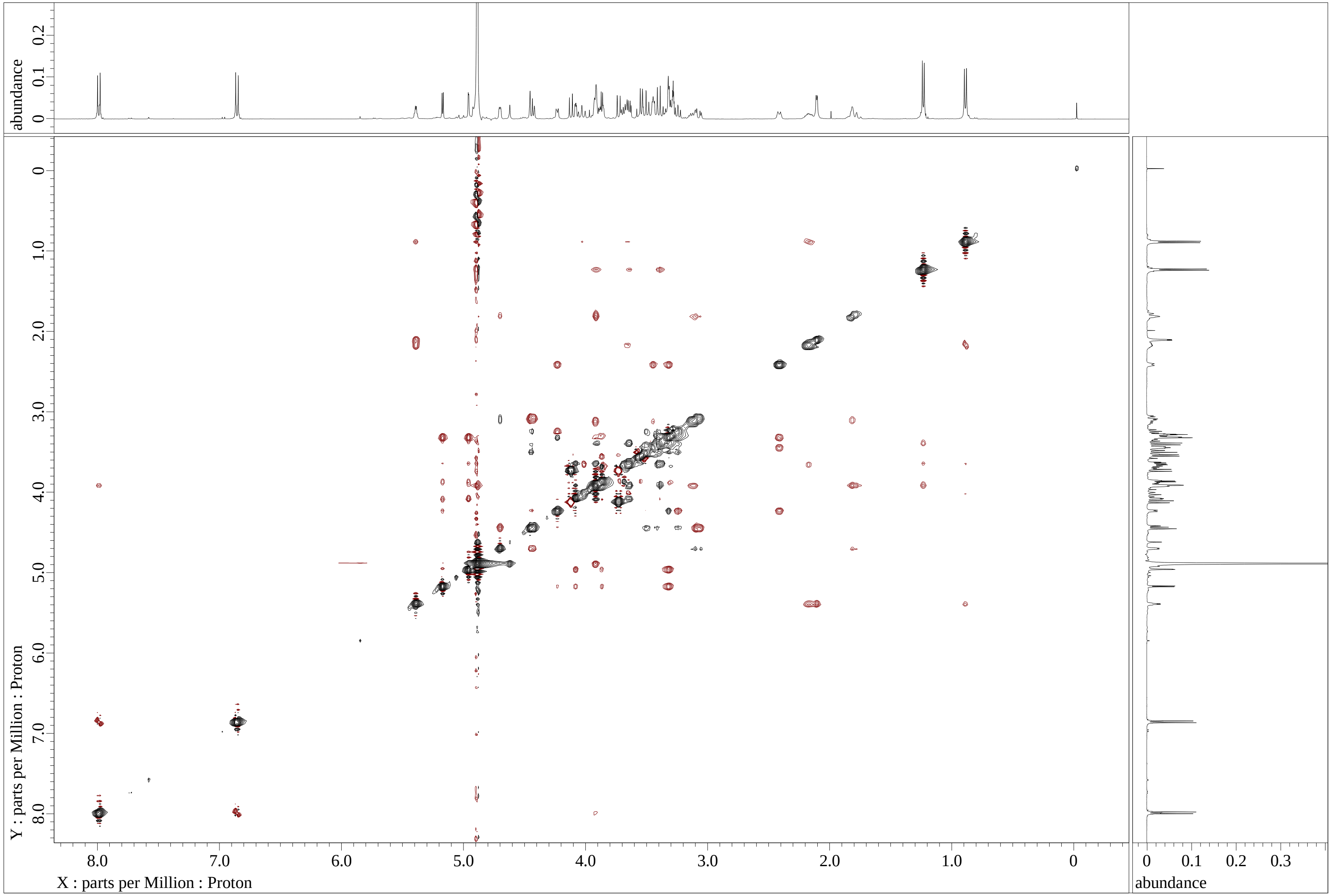

Figure S18 2D ROESY spectrum of **2** in CD_3OD

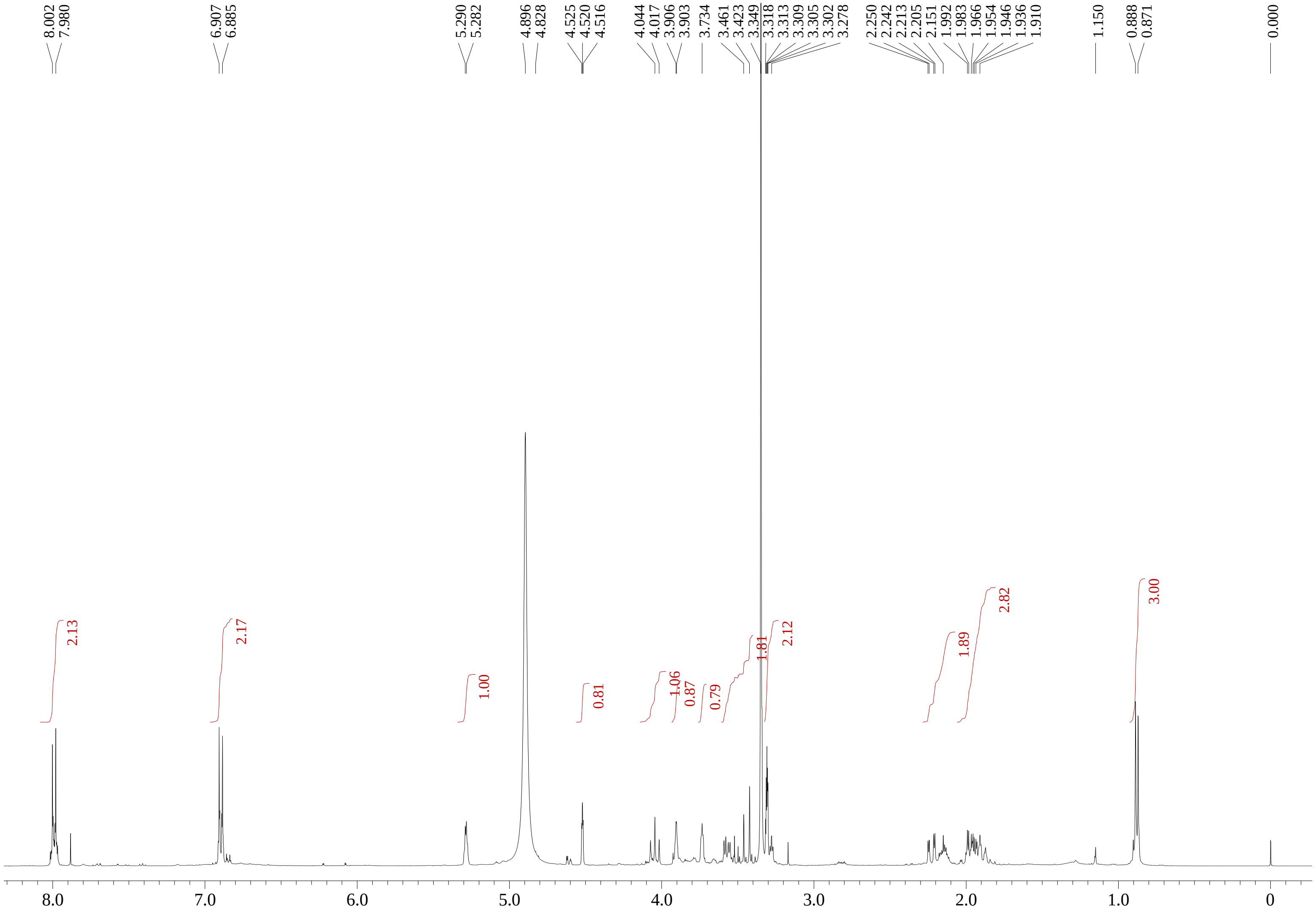

Figure S19 ^{1}H NMR spectrum of **3** in CD_3OD

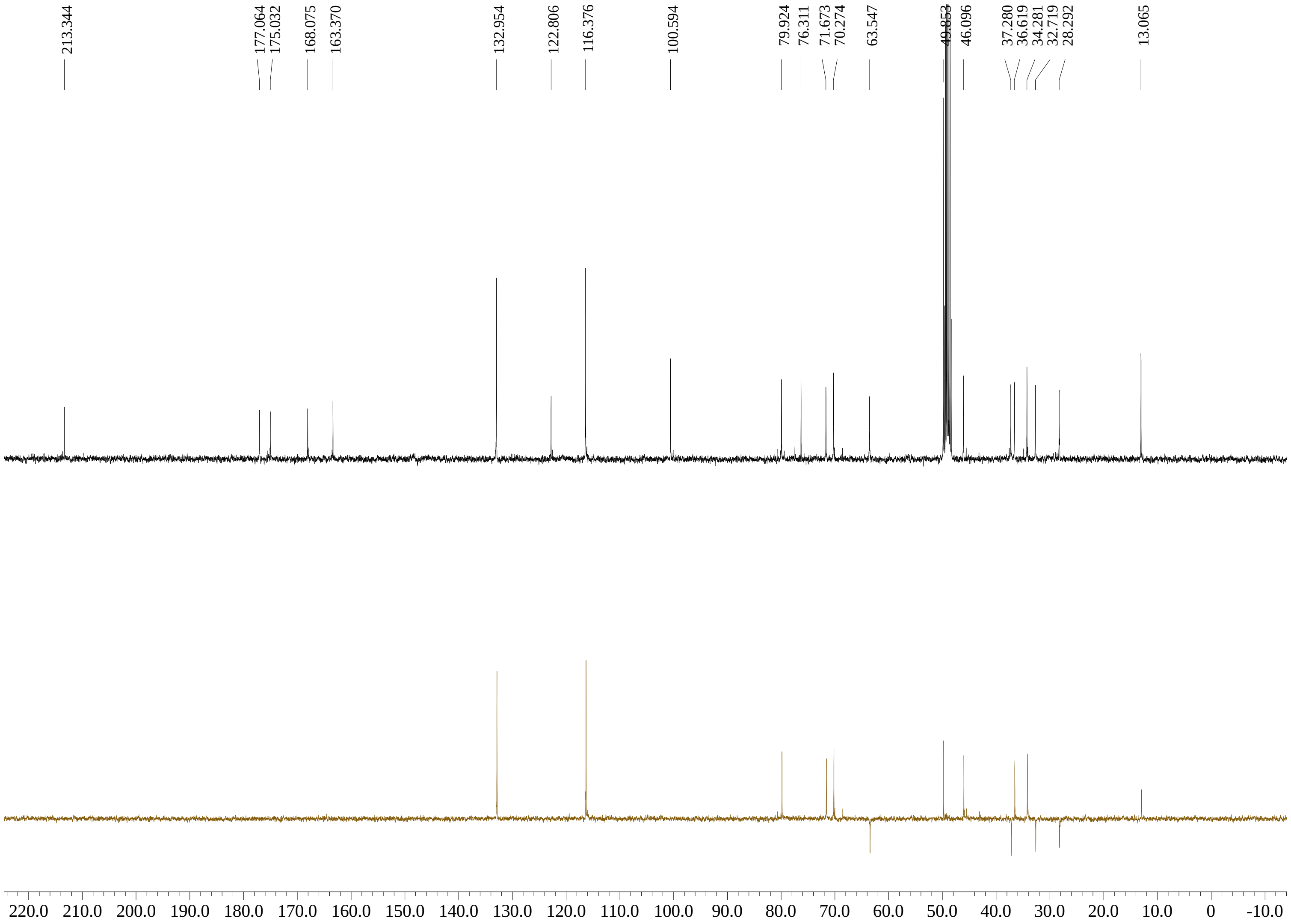

Figure S20 ^{13}C NMR and DEPT135 spectra of **3** in CD_3OD

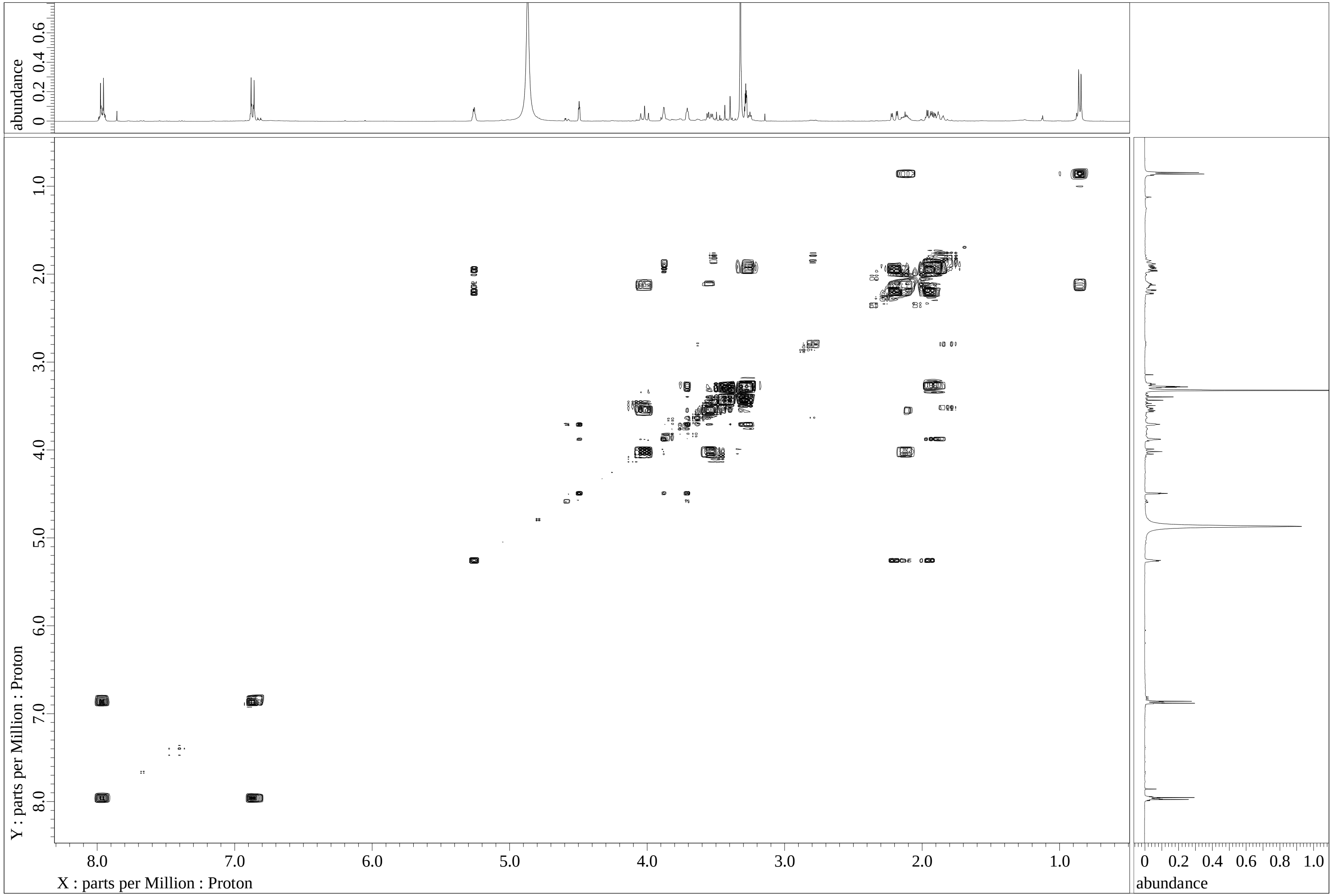

Figure S21 DQF-COSY spectrum of **3** in CD_3OD

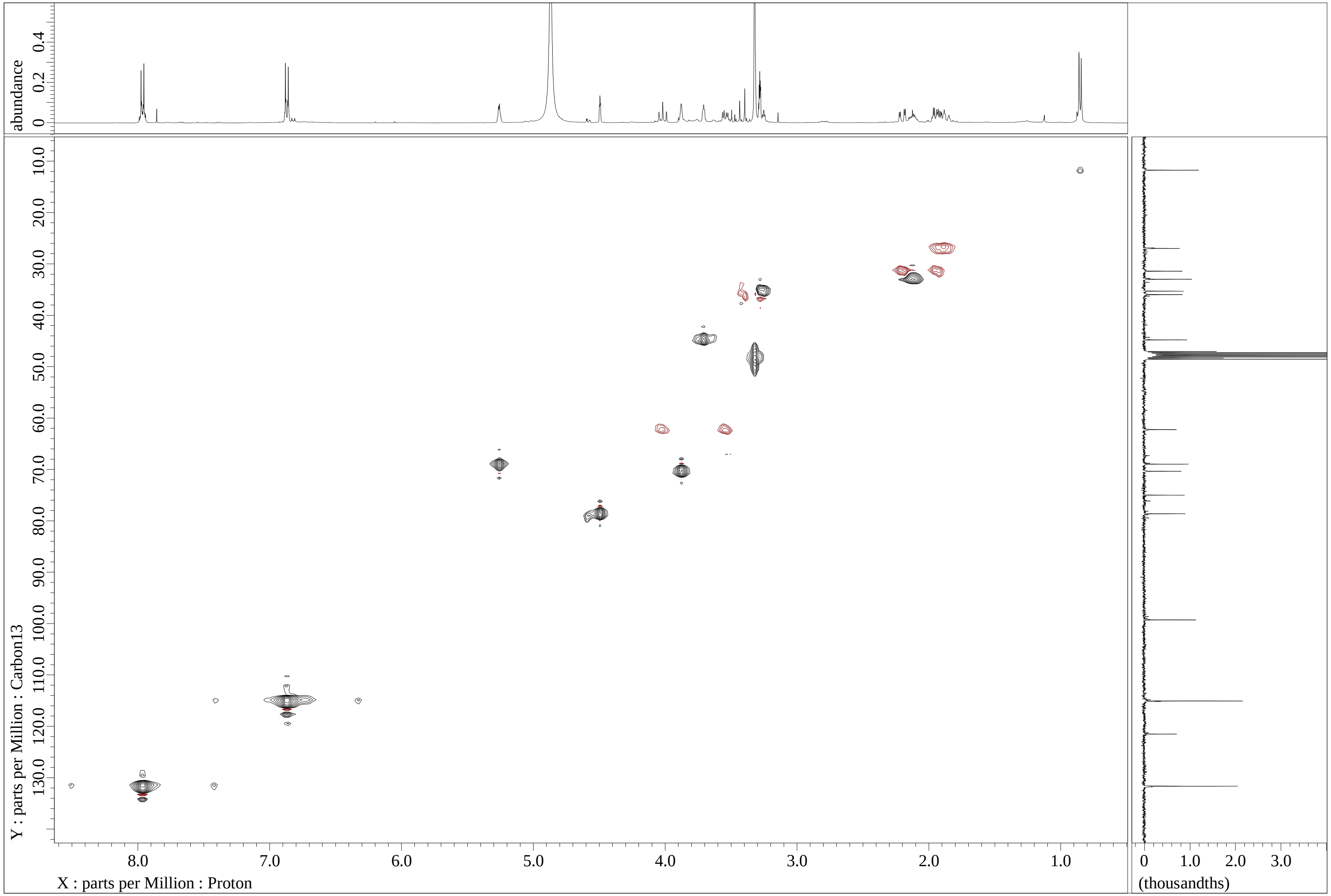

Figure S22 HSQC spectrum of **3** in CD_3OD

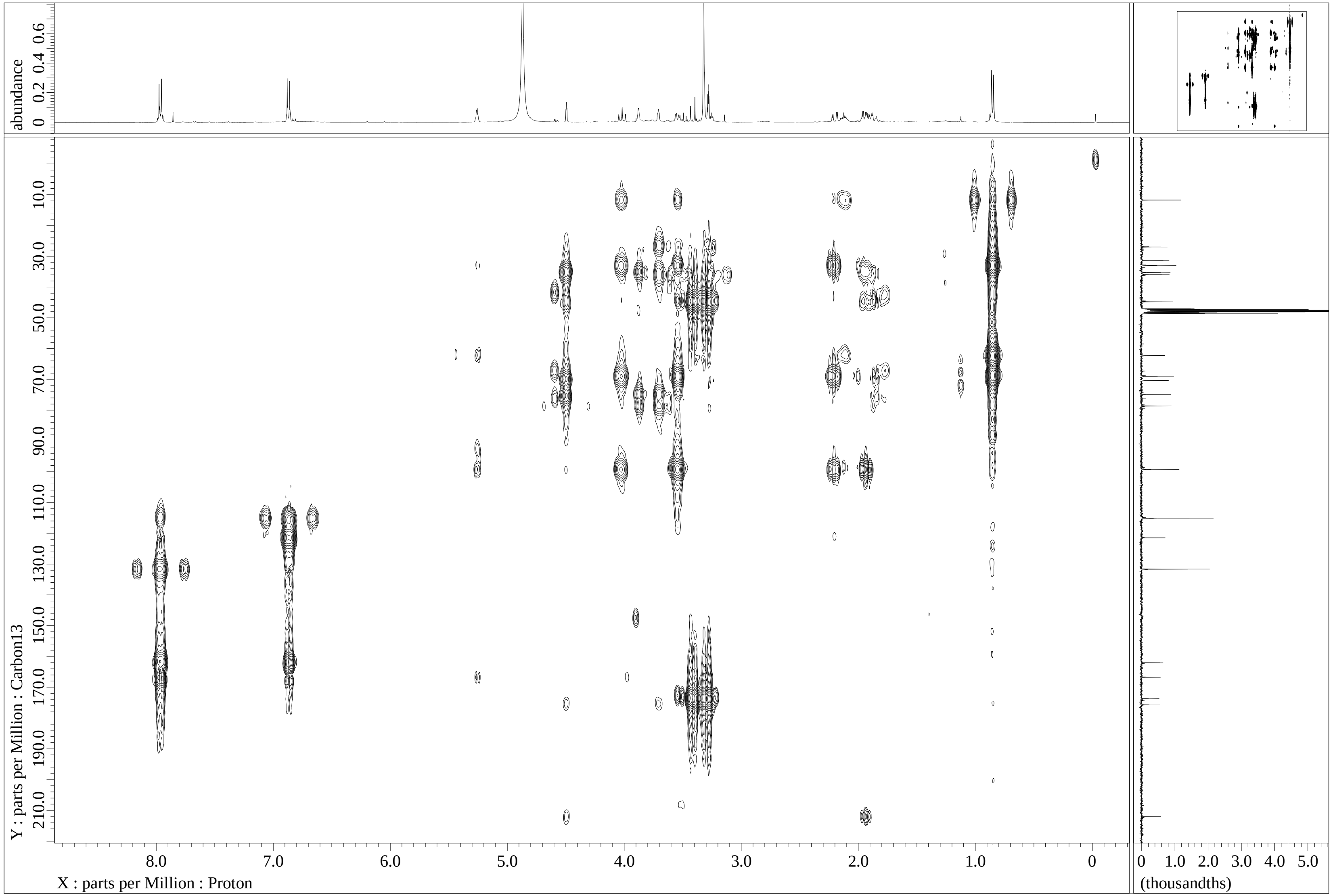

Figure S23 HMBC spectrum of **3** in CD_3OD

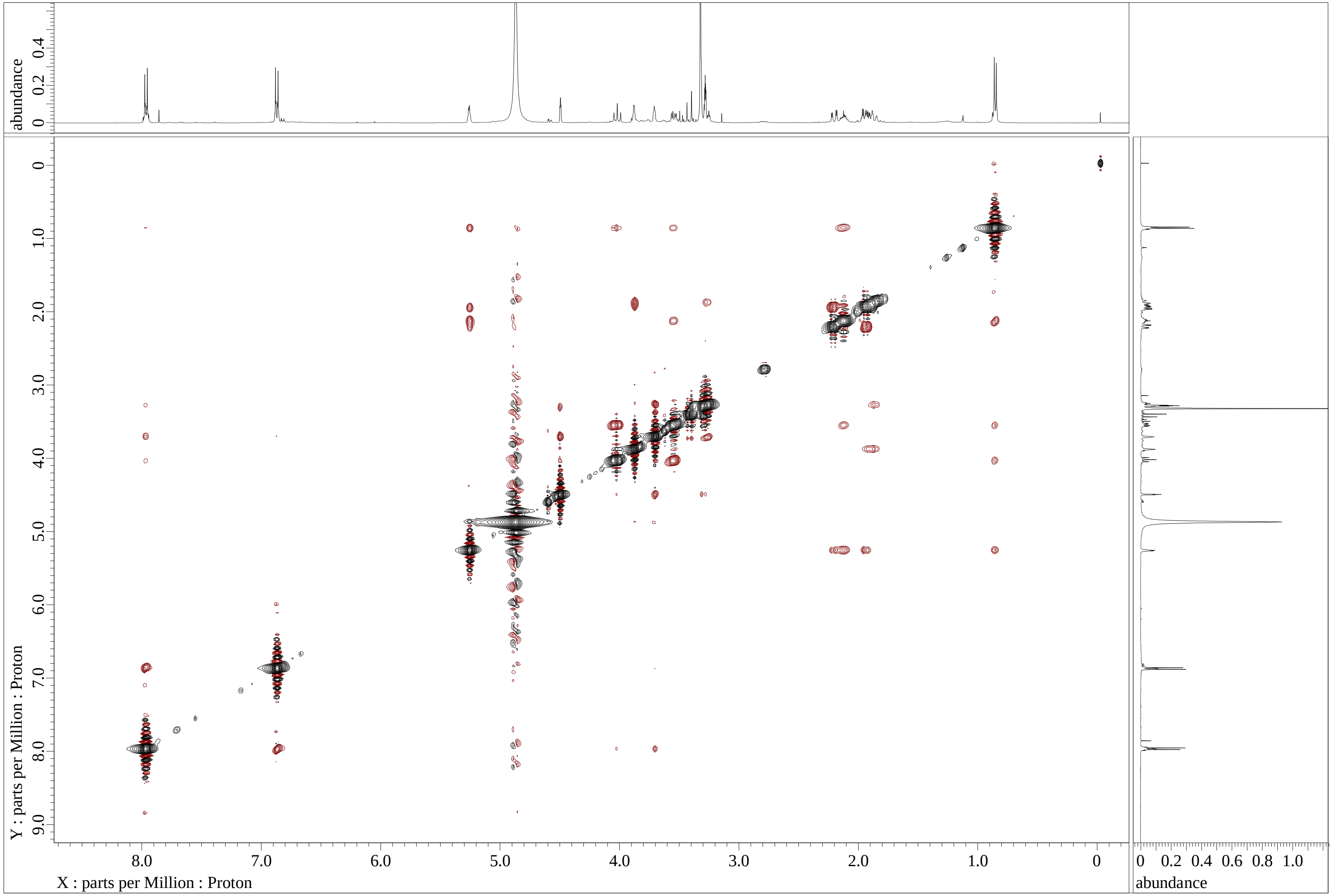

Figure S24 NOESY spectrum of **3** in CD_3OD

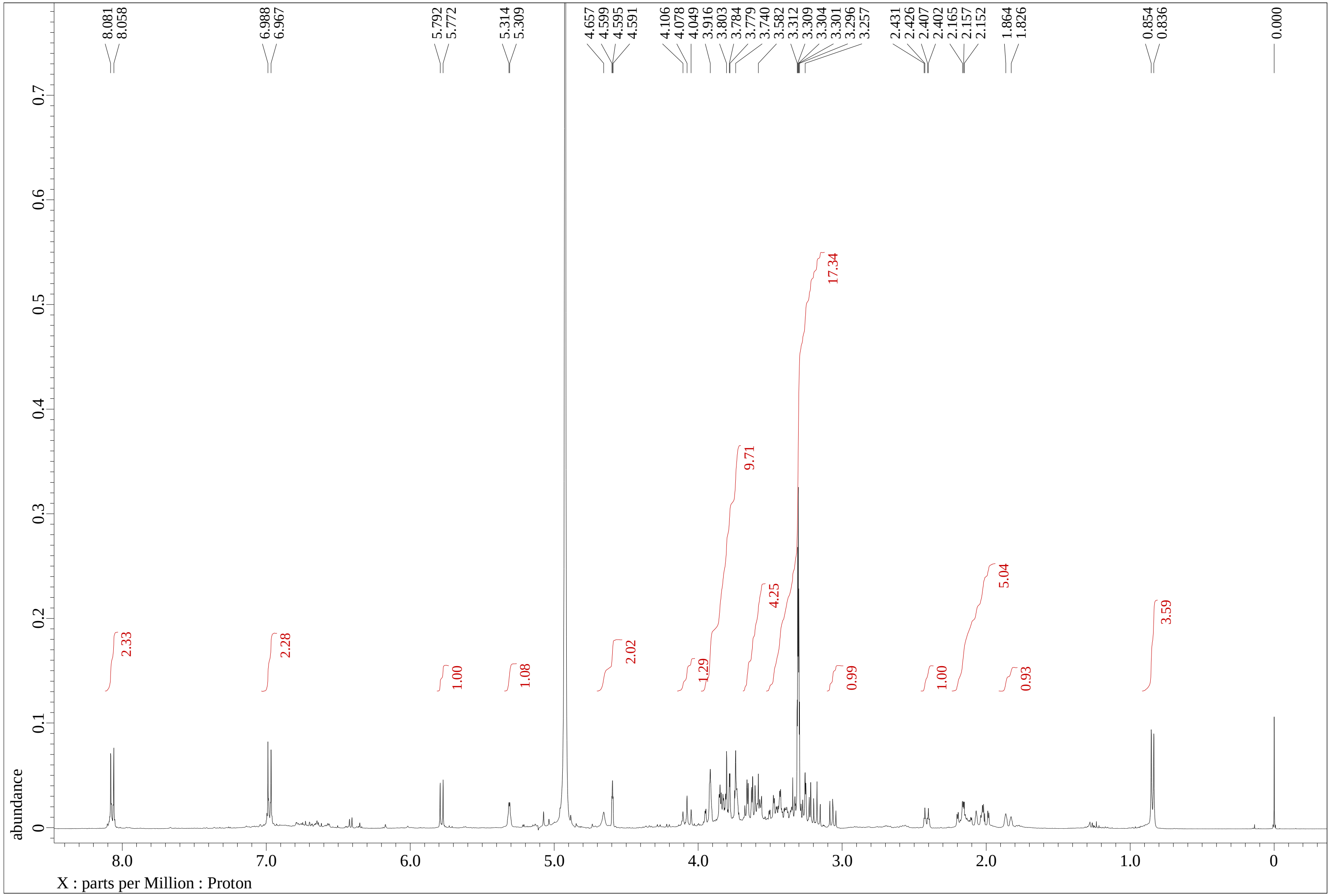

Figure S25 ^{1}H NMR spectrum of **4** in CD_3OD

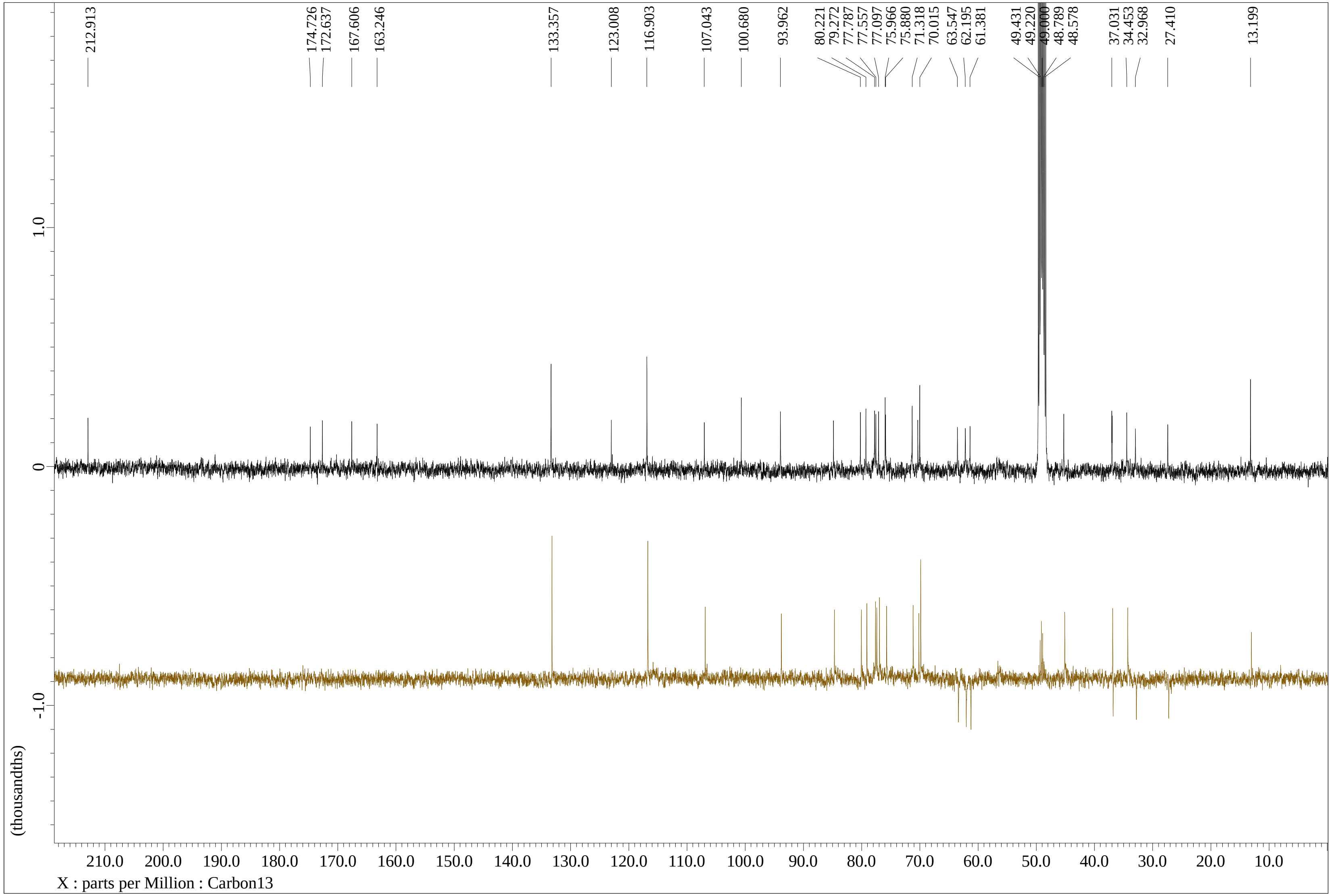

Figure S26 ^{13}C NMR and DEPT135 spectra of **4** in CD_3OD

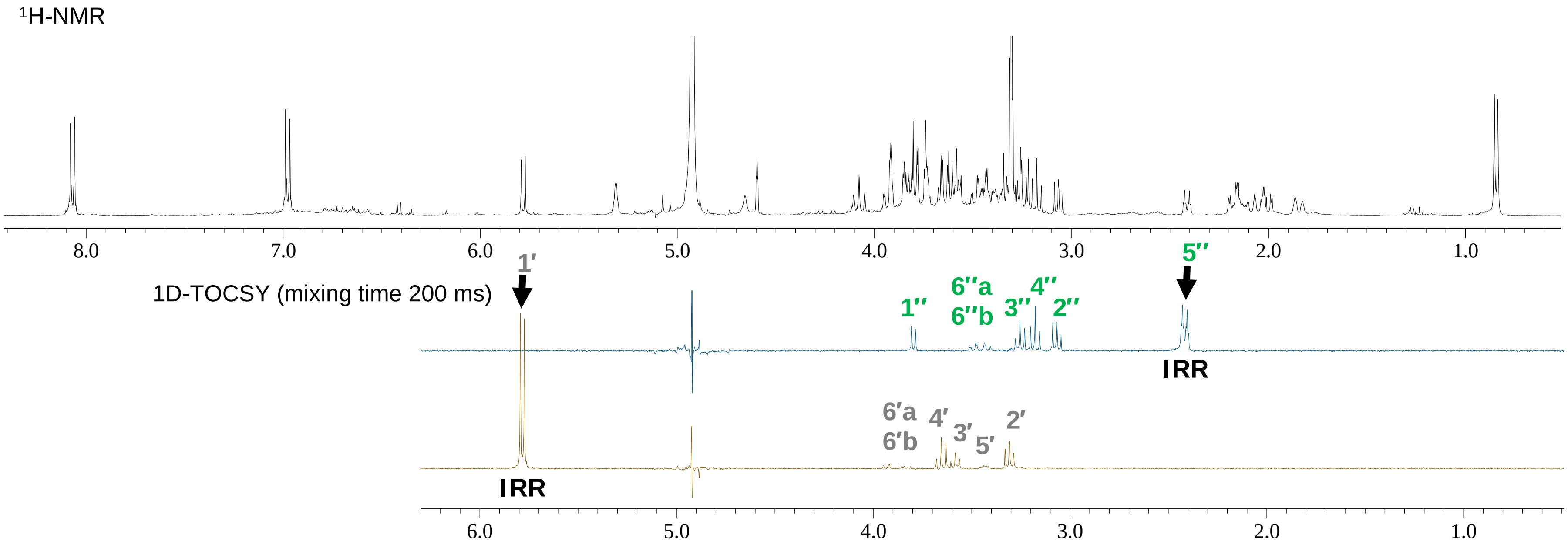

Figure S27 1D-TOCSY spectra of **4** in CD_3OD

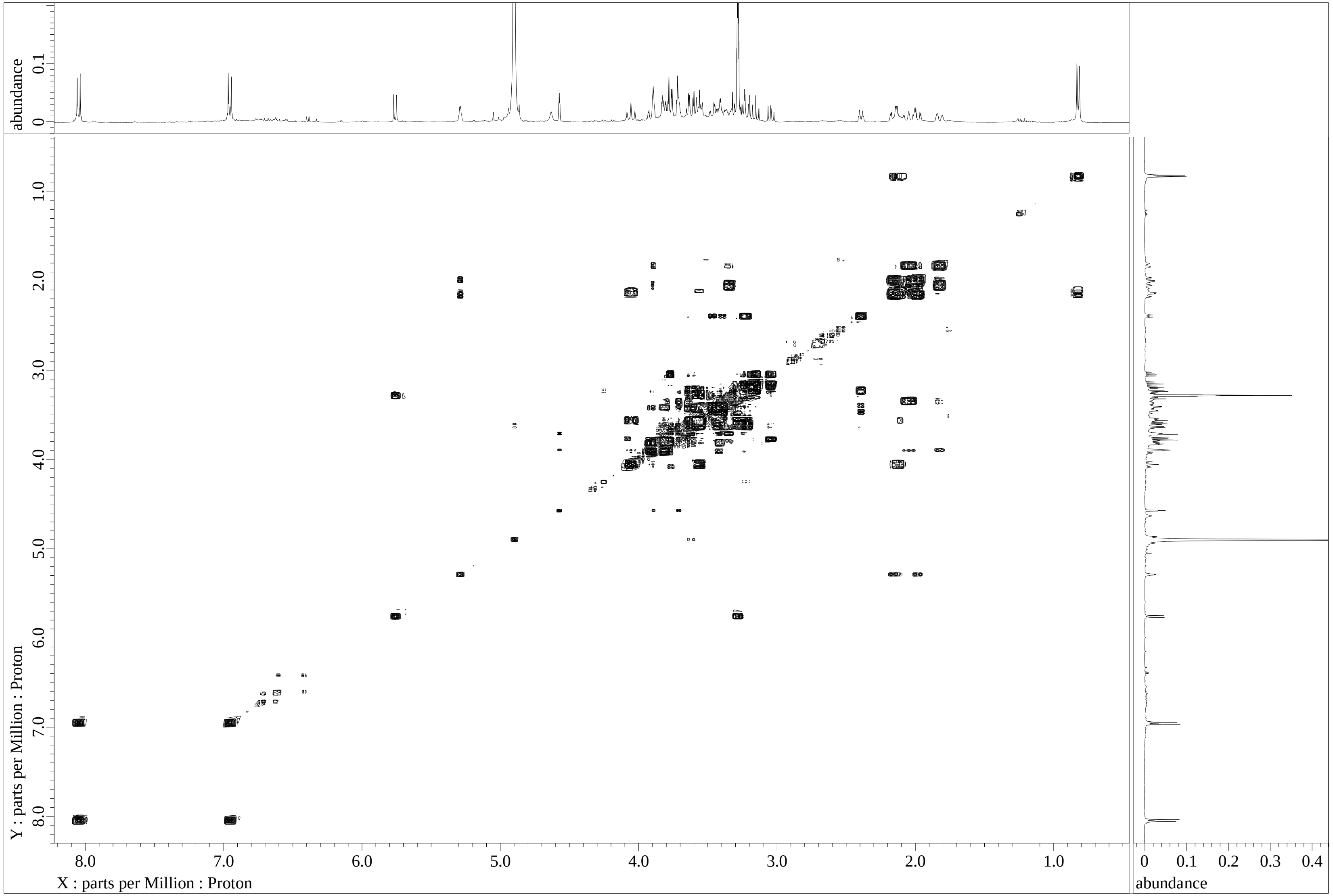

Figure S28 DQF-COSY spectrum of **4** in CD_3OD

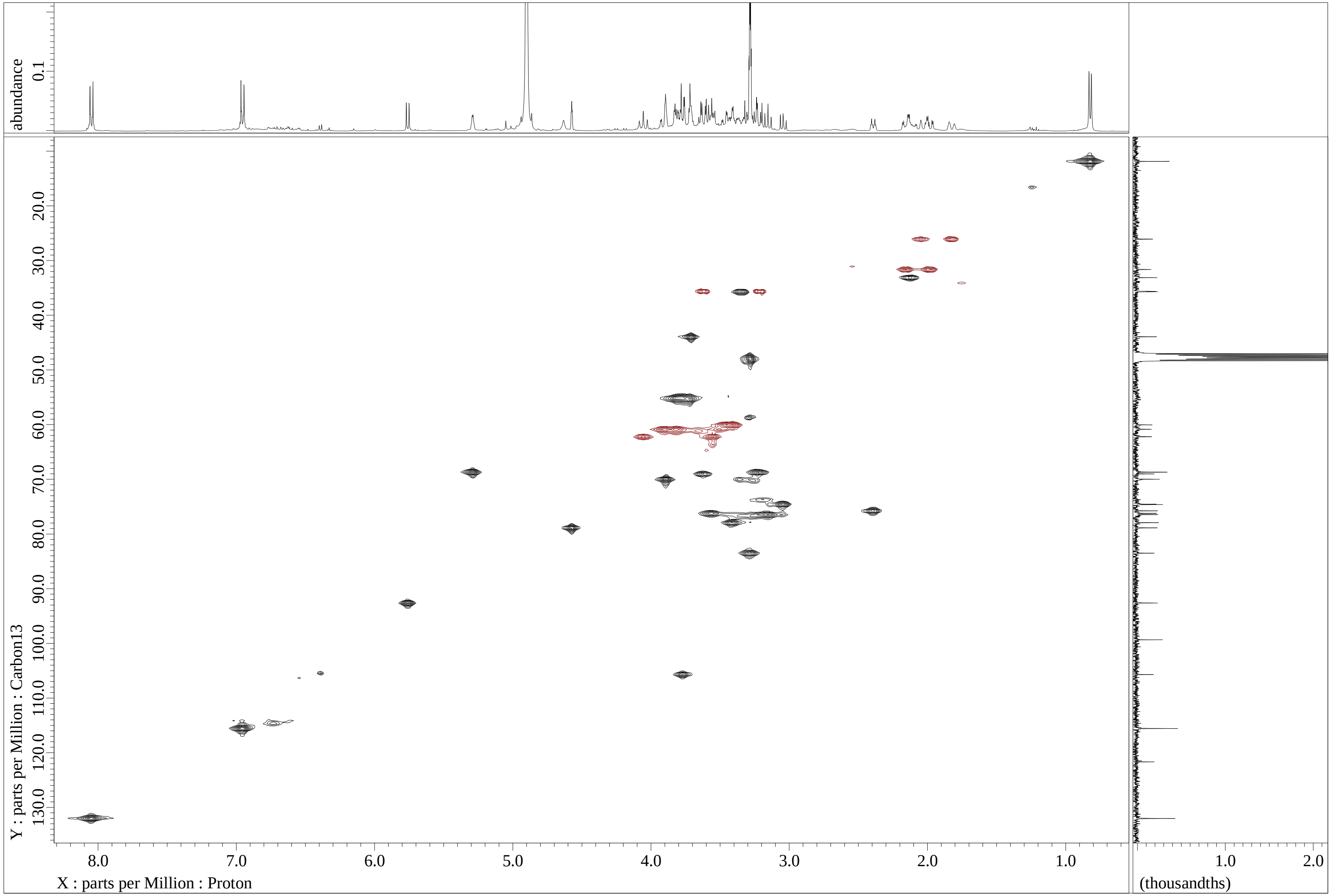

Figure S29 HSQC spectrum of **4** in CD_3OD

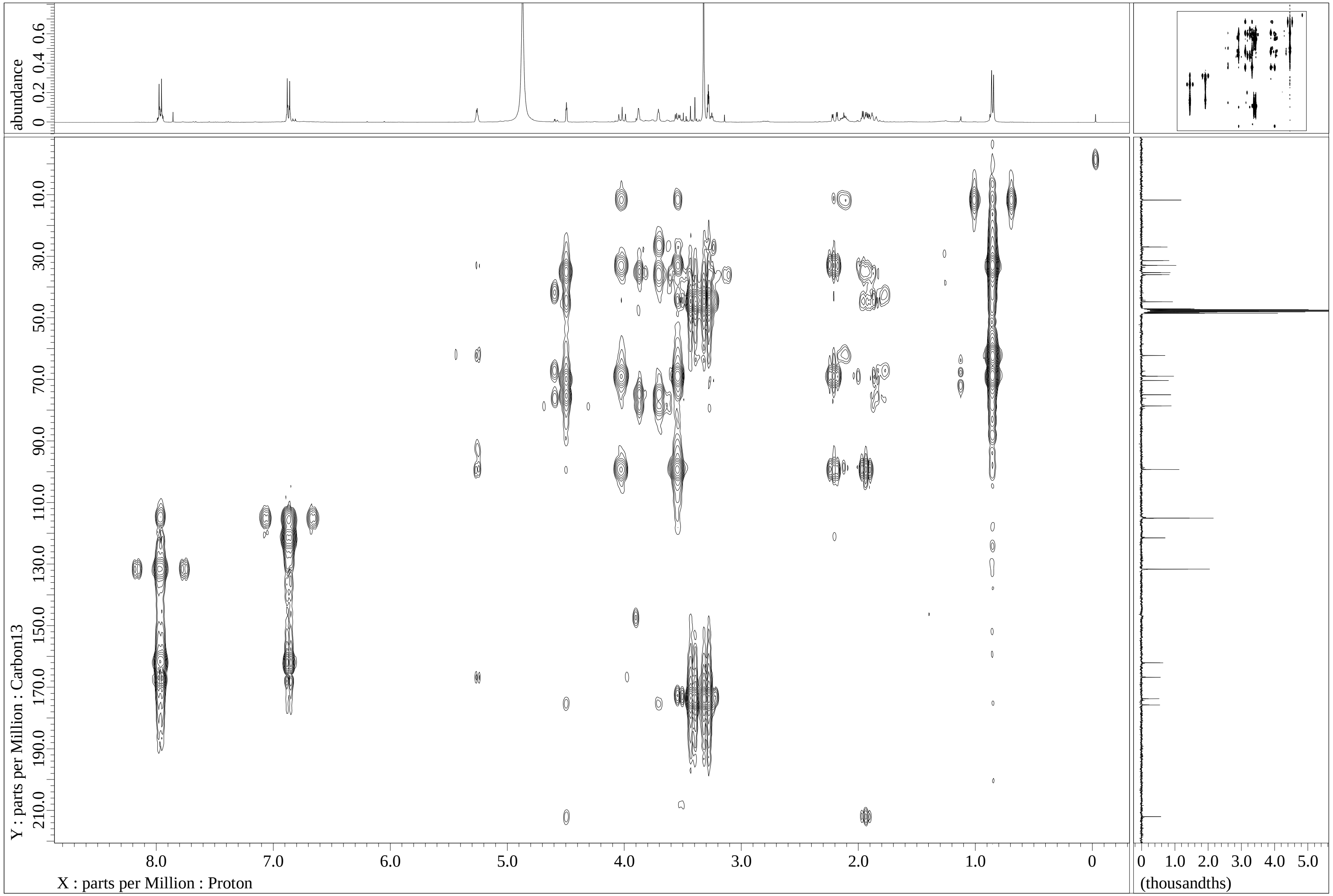

Figure S30 HMBC spectrum of **3** in CD_3OD

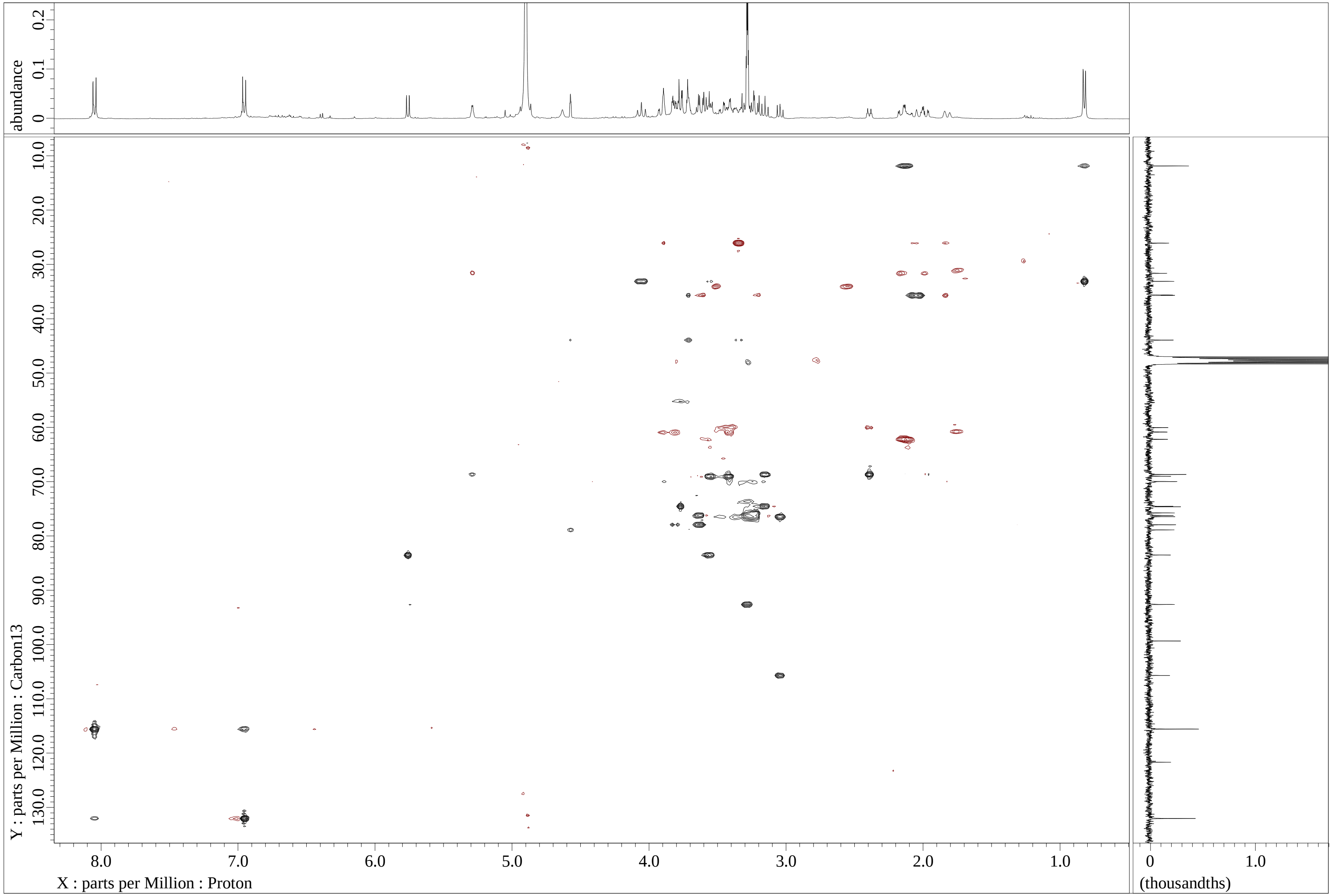

Figure S31 H2BC spectrum of **3** in CD_3OD

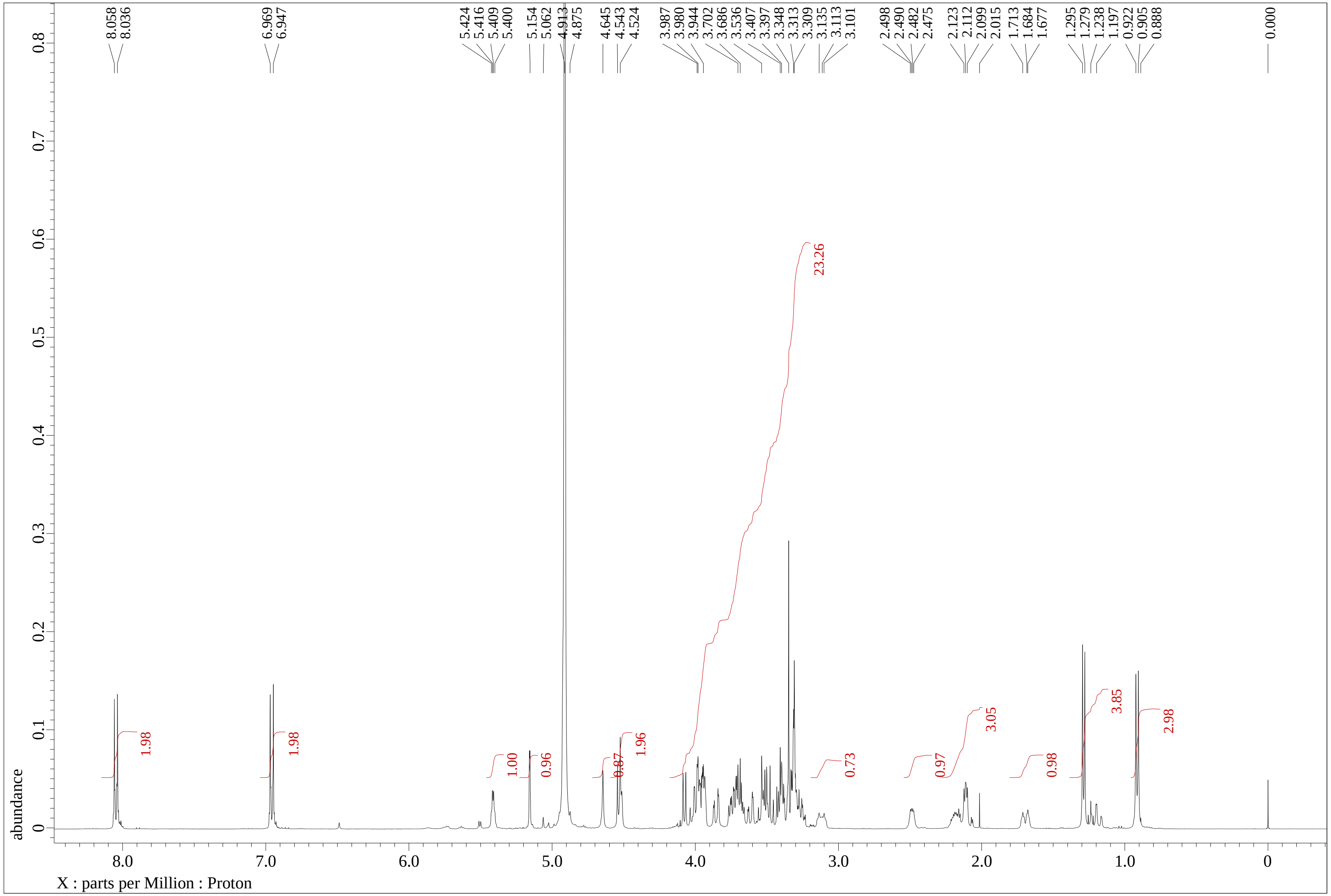

Figure S32 ^{1}H NMR spectrum of **6** in CD_3OD

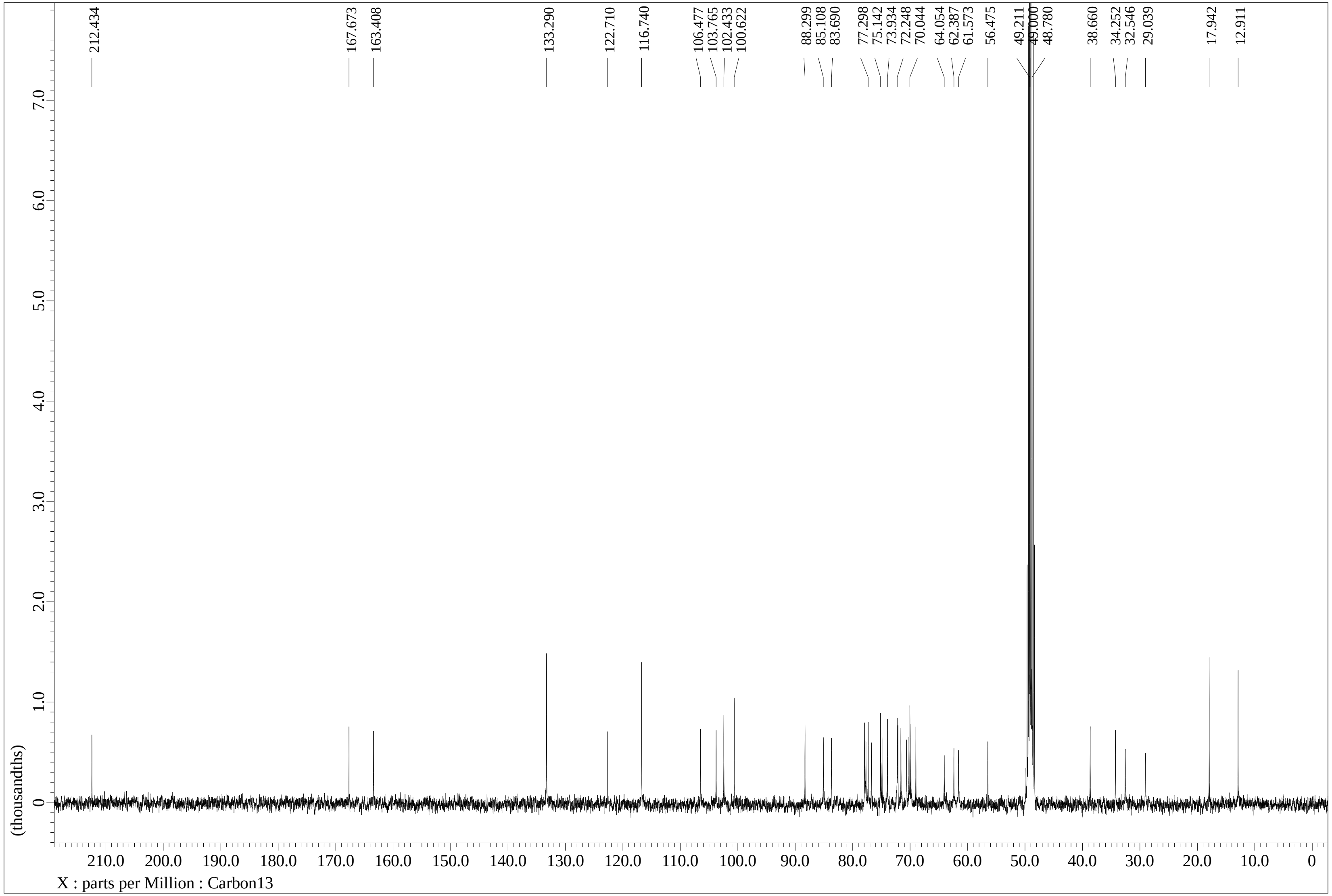

Figure S33 ^{13}C NMR spectrum of **6** in CD_3OD

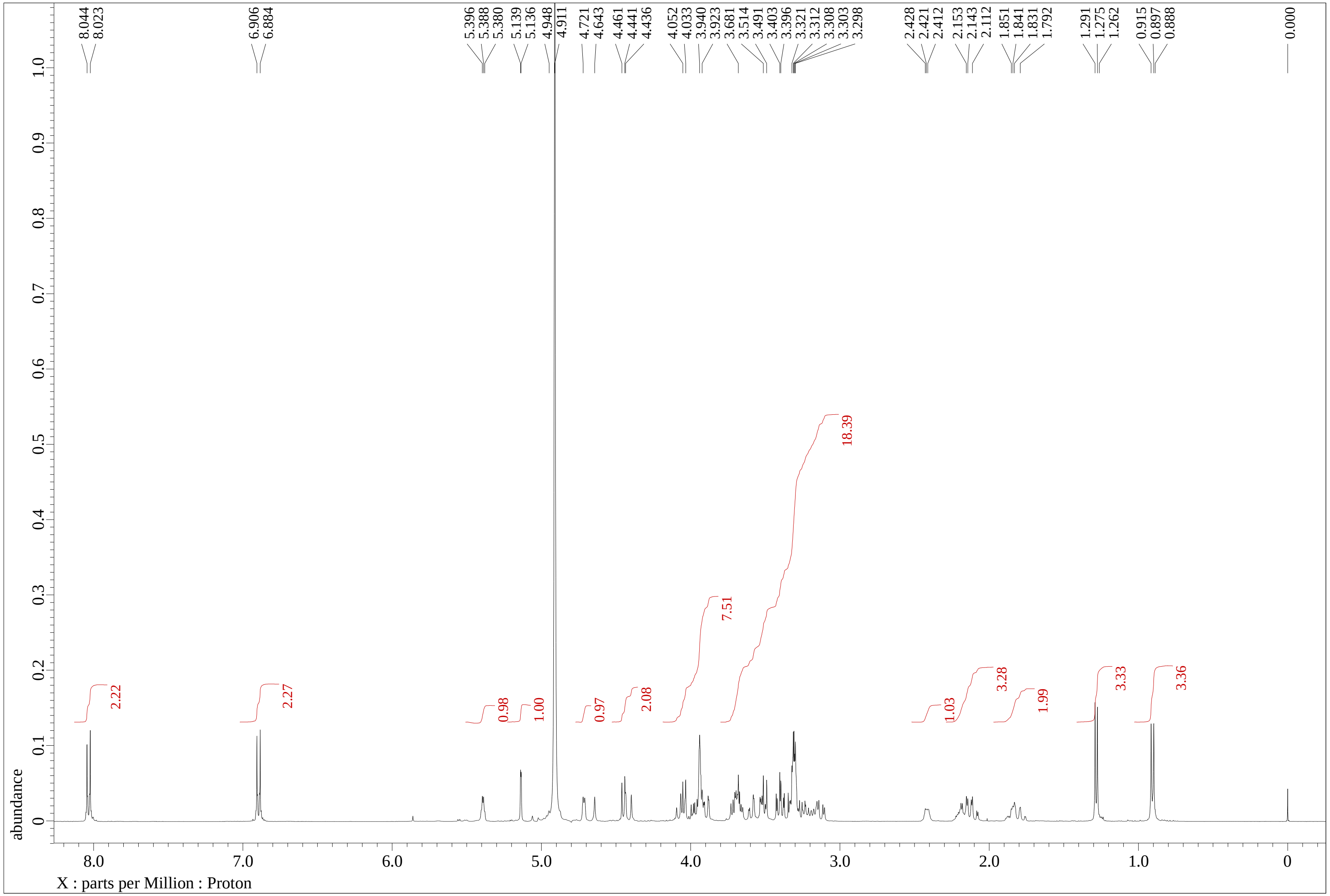

Figure S34 ^{1}H NMR spectrum of **7** in CD_3OD

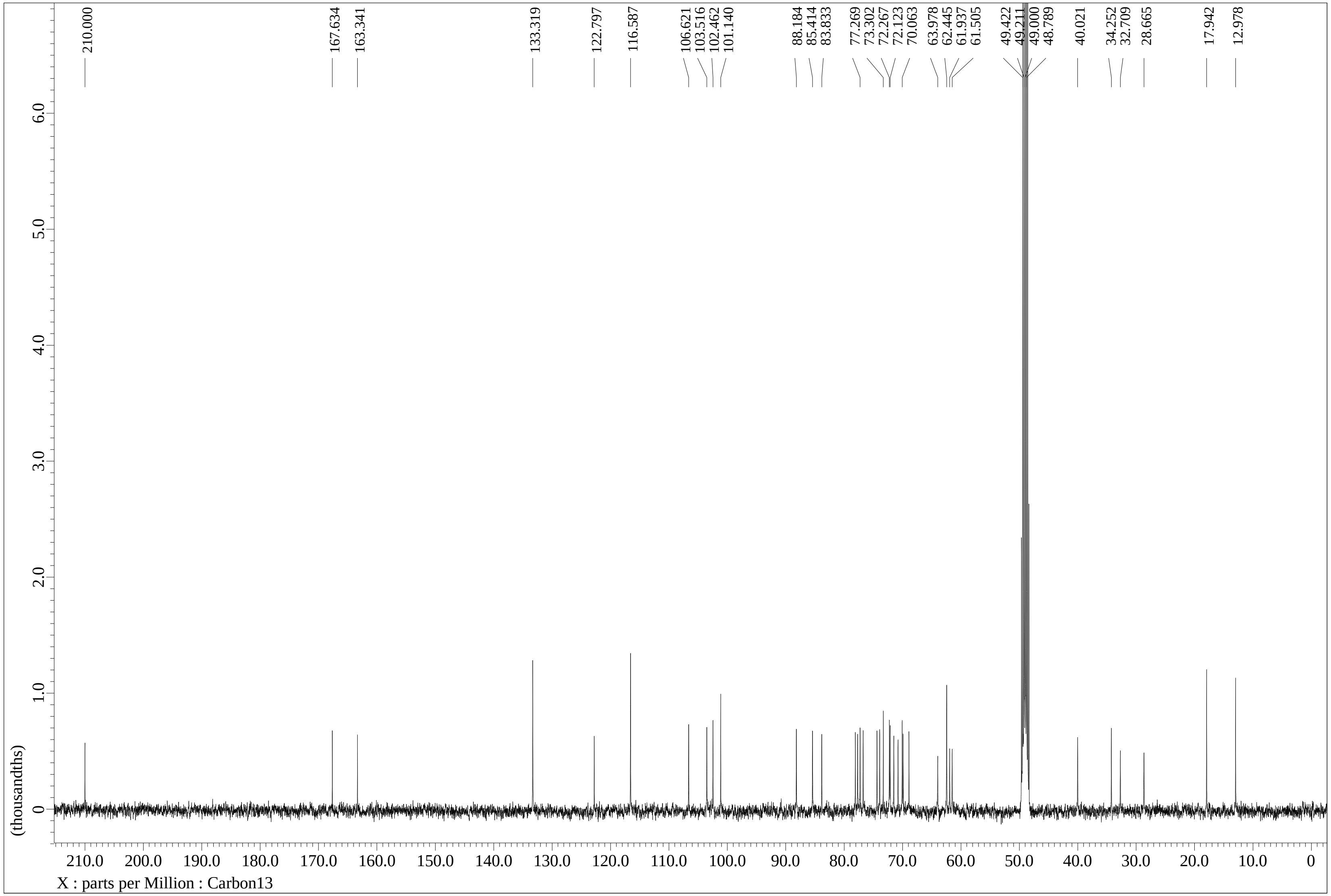

Figure S35 ^{13}C NMR spectrum of **7** in CD_3OD

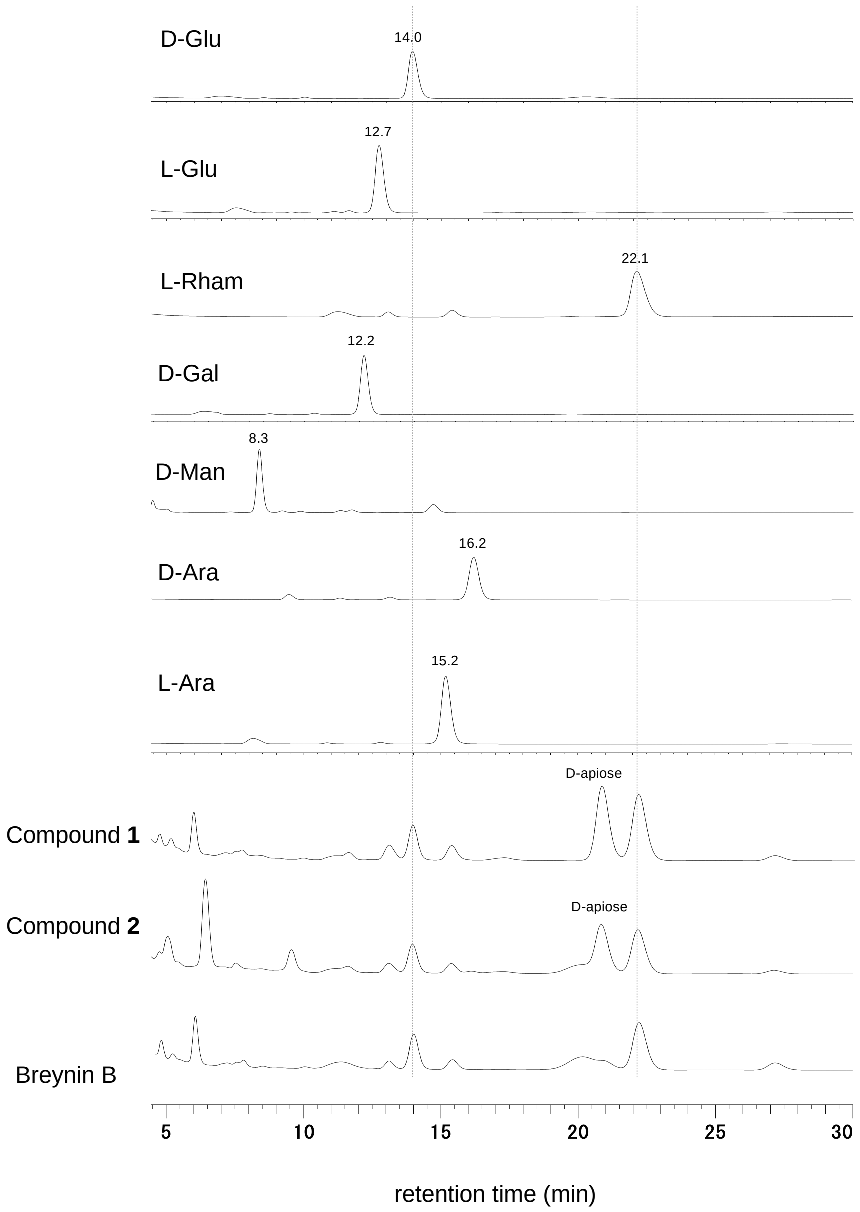

Figure S36 HPLC chromatograms for sugar identification
